# Surveillance of the Genetic Signature in Circulating Tumor DNA for Guiding Adjuvant Chemotherapy in Urothelial Carcinoma: Protocol for a Pilot Randomized Controlled Trial

**DOI:** 10.2196/72597

**Published:** 2025-08-26

**Authors:** Yongle Zhan, Xiaohao Ruan, Yishuo Wu, Tsun Tsun Stacia Chun, Chi Yao, Ruofan Shi, Jiacheng Liu, Salida Ali, Ruochen Ma, Da Huang, Yi Gao, Ying Xu, Lu Chen, Qijun Du, Ada Tsui-Lin Ng, Cho Wing Bryan Li, Danfeng Xu, Rong Na

**Affiliations:** 1 Department of Surgery LKS Faculty of Medicine The University of Hong Kong Hong Kong China (Hong Kong); 2 Department of Urology Ruijin Hospital Shanghai Jiao Tong University School of Medicine Shanghai China; 3 Department of Urology Huashan Hospital Fudan University Shanghai China; 4 Department of Clinical Oncology Pamela Youde Nethersole Eastern Hospital Hong Kong China (Hong Kong); 5 Division of Urology Department of Surgery Queen Mary Hospital Hong Kong China (Hong Kong); 6 Department of Medicine Queen Mary Hospital Hong Kong China (Hong Kong)

**Keywords:** urothelial carcinoma, adjuvant chemotherapy, circulating tumor DNA, sequencing, molecular residual disease, precision medicine, randomized controlled trial

## Abstract

**Background:**

Urothelial carcinoma is one of the most commonly diagnosed cancers worldwide, with a poor 5-year survival rate. As genomics is the backbone of the precision medicine paradigm, the genetic signature in circulating tumor DNA (ctDNA) is emerging as a pivotal biomarker for detecting early-stage cancer and molecular residual disease (MRD).

**Objective:**

We aim to evaluate the feasibility and preliminary effects of a ctDNA-based sequencing approach for detecting MRD and guiding adjuvant chemotherapy in postoperative urothelial carcinomas.

**Methods:**

We will perform a stratified 2-arm pilot randomized controlled trial in 2 tertiary hospitals in Hong Kong, involving patients with urothelial carcinomas (pT2-4a N0-2 M0) undergoing radical resection. We plan to recruit 20 patients and determine stratification according to their MRD status before randomization. Patients in each stratum (MRD-positive and MRD-negative groups) will be randomly allocated to either a 4-cycle gemcitabine plus cisplatin chemotherapy arm or a standard management arm in a 1:1 ratio. ctDNA MRD will be tested using a personalized next-generation sequencing panel, which is designed based on the individual’s whole exome sequencing results from the operation specimen. The primary outcome is the feasibility of this trial (ie, recruitment, retention, adherence, and completeness). The secondary outcome is treatment-related adverse events. Exploratory outcomes include radiographic disease-free survival, cancer-specific survival, overall survival, ctDNA clearance in patients with ctDNA MRD-positive status, quality of life, fear of cancer recurrence, and cost-effectiveness. Benchmarks for feasibility evaluation are set as (1) ≥20% recruitment response rate, (2) ≤20% loss to follow-up or withdrawal, (3) ≥80% intervention adherence, and (4) ≤20% missing value rate. Each benchmark will be assigned one score, and a total score of 4, 2 to 3, and 0 to 1 will be deemed high, medium, and low feasibility, respectively. Safety evaluations will be presented as numbers and proportions of the adverse events. ANOVA and the Kruskal-Wallis test will be used for continuous outcome variables, whereas the chi-square test and the Fisher exact test will be used for categorical outcome variables. Hazard ratios will be calculated to compare the preliminary treatment effect of the gemcitabine plus cisplatin arm against the standard management arm on survival within each MRD group.

**Results:**

This project was funded in February 2024. Patient recruitment started on May 2, 2024. Recruitment and data collection for the trial are ongoing. Data analysis will be performed in mid-2025 and the results of this study are expected to be published in late 2025.

**Conclusions:**

Genetic signature in ctDNA is informative for personalized management of postoperative urothelial carcinomas, including personalized treatment and early detection of disease progression.

**Trial Registration:**

ClinicalTrials.gov NCT06257017; https://clinicaltrials.gov/study/NCT06257017

**International Registered Report Identifier (IRRID):**

DERR1-10.2196/72597

## Introduction

### Background

Urothelial carcinomas, located in the upper (pyelocaliceal cavities and ureter) or lower (bladder and urethra) urinary tract [1], are one of the most commonly diagnosed cancers worldwide [2]. Although radical resection (ie, cystectomy and nephroureterectomy) is the established therapy for muscle-invasive urothelial carcinoma (MIUC), patients who have undergone surgery still have a poor prognosis with an estimated 5-year disease-specific survival rate of 50% [3]. To improve the overall survival (OS) and reduce the recurrence risk of MIUC, perioperative chemotherapy is recommended as a standard of care [4]. However, it remains a challenge to determine the presence of residual tumor cells. Adjuvant chemotherapy may cause overtreatment and unnecessary toxicity in patients without molecular residual disease (MRD) [5]. In contrast, those with residual disease may miss the potentially beneficial treatment by the time relapse is detected by imaging. Another challenging issue is that, despite clinical guidelines, neoadjuvant chemotherapy (preoperative) and adjuvant chemotherapy (postoperative) are not commonly or regularly provided in Hong Kong (about 6% and 50%, respectively, based on medical records in our center from the past 5 years) due to potential side effect concerns by both physicians and patients. Therefore, it is pivotal to distinguish patients with residual disease from those with early relapse to help physicians prescribe a precise treatment regimen.

As genomics is the backbone of the precision medicine paradigm, the genetic signature in circulating tumor DNA (ctDNA) is emerging as a pivotal biomarker for detecting early-stage cancer and MRD [6]. MRD, a potential indicator of clinical tumor relapse, can be identified by ctDNA in peripheral blood, with a lead time of several months earlier than that of radiological detection [7]. Methods for ctDNA MRD detection can be divided into various categories: (1) those based on techniques, including polymerase chain reaction (PCR) and next-generation sequencing (NGS); (2) those based on assay panel size, including single-locus or multiplexed assays, targeted sequencing, and untargeted sequencing (whole genome or whole exome sequencing [WES]); and (3) those based on previous knowledge of patient-specific tumor mutational profiles, such as tumor-agnostic assays and tumor-informed assays [8-10].

Several clinical validation studies have confirmed that ctDNA is related to recurrence with high sensitivity (>80%) and specificity (>90%) [11]. For instance, 94% of patients with lung cancer and detectable ctDNA in their first posttreatment blood samples were subsequently identified to have recurrence, with an observed lead time of 5.2 months [12]. Similarly, ctDNA detection has been reported to precede radiographic progression in breast, colorectal, pancreatic, and bladder carcinomas by a median of 9.5, 8.7, 4.2, and 2.8 months [13-16]. In addition, ctDNA, with a half-life shorter than 2 hours in the plasma, is an optimal surrogate for real-time relapse monitoring [7]. Thus, ctDNA MRD can possibly serve as a cancer-agnostic surrogate analyte for risk stratification of tumor recurrence [6], thereby guiding individually tailored treatments.

In our preliminary investigation, we used a WES technique to detect the genetic signatures of 3 bladder cancer samples. As shown in Multimedia Appendix 1, a total of 98, 481, and 120 variants with different mutation types were detected in the 3 individuals, respectively. Personalized genetic signatures have been used to design assay panels to dynamically monitor bloodstream ctDNA MRD. We found that ctDNA MRD could precisely reveal the disease severity at baseline, reflect a disease-free status after treatment, and indicate a potential relapse after follow-up for several months (Multimedia Appendix 2). Given the success of our preliminary investigation and the strengths of its noninvasiveness and superior sensitivity, ctDNA MRD can possibly serve as a cancer-agnostic surrogate analyte for risk stratification of tumor recurrence, thereby guiding individually tailored treatments.

Increasing evidence has evaluated the clinical utility of a ctDNA-oriented approach for adjuvant chemotherapy. A multicenter randomized controlled trial (RCT) [17] of a ctDNA-guided approach for postsurgical treatment of colon cancer showed that patients in the ctDNA-guided group received lower adjuvant chemotherapy (15% vs 28%) without compromising disease-free survival (DFS) compared with the standard management (SM) group (2-year DFS absolute difference 1.1%, 95% CI −4.1% to 6.2%). Another randomized trial of a treatment for female breast cancer [18] also indicated that a genomically directed therapy could reduce the uptake of capecitabine and was noninferior to an empirical treatment. As to the utility in MIUC, a post hoc analysis from a multicenter randomized trial [19] revealed that in the ctDNA MRD-positive group, patients receiving adjuvant atezolizumab treatment had improved DFS compared with those receiving observation (median DFS 5.9 months vs 4.4 months; median OS 25.8 months vs 15.8 months), whereas in the ctDNA MRD-negative group, there was no difference in clinical outcomes between treatment and observation arms. A biomarker analysis from another clinical trial [20] demonstrated that the dynamic changes in ctDNA during the perioperative period (proportion of ctDNA positive: 63%, 47%, and 14% at baseline, postneoadjuvant, and postcystectomy time points, respectively) could potentially guide clinical monitoring and postsurgical treatment.

However, there is limited evidence from clinical trials on the clinical efficacy of ctDNA MRD detection in guiding treatment regimens for patients who have undergone surgery for urothelial cancer, particularly among the Chinese population. Thus, we propose a pilot RCT, where patients will be stratified by ctDNA results and randomly allocated to receive either 4 cycles of gemcitabine plus cisplatin (GC) chemotherapy or SM regimens in a 1:1 ratio. In this study, we hypothesize that an improved DFS will be observed with GC chemotherapy administration among patients with ctDNA MRD-positive status, whereas there will be no benefit among patients with ctDNA MRD-negative status. In addition, the genetic profile of ctDNA for detecting MRD can provide real-time recurrence monitoring with a significant lead time before radiological relapse.

### Objectives

We aim to (1) assess the predictive value of a ctDNA-guided MRD profiling approach for postoperative adjuvant chemotherapy, (2) assess the clinical approach of MRD surveillance by serial ctDNA testing in patients with ≥T2 urothelial cancer who have undergone surgery, and (3) provide preliminary data to assess the feasibility of a definitive trial and perform a more accurate sample size estimation in a future phase 3 clinical trial.

## Methods

### Study Design, Setting, and Recruitment

A multicenter, open-label pilot RCT is proposed in the Queen Mary Hospital and the Pamela Youde Nethersole Eastern Hospital. Eligible patients with histologically confirmed MIUC will be invited to join the study from February 19, 2024, to February 18, 2025, in the Queen Mary Hospital and the Pamela Youde Nethersole Eastern Hospital in Hong Kong. The study and informed consent process will be explained to all invited patients before they sign the written consent form to participate in this study.

### Ethical Considerations

This study has been approved by the Central Institutional Review Board of the Hospital Authority in Hong Kong (CIRB-2023-189-2) on February 28, 2024. Written informed consent will be obtained from all study participants. Participants will be informed of confidentiality and their right to leave the study at any time and without any penalty. Data are deidentified. This study conforms to the principles outlined in the Declaration of Helsinki and is registered on ClinicalTrials.gov (NCT06257017).

### Eligibility Criteria

The participants will be recruited based on the predefined inclusion and exclusion criteria, as presented in Textbox 1.

**Inclusion and exclusion criteria.**

**Inclusion criteria**

- Aged 18 to 70 years
- A score of ≤1 for the Eastern Cooperative Oncology Group performance status
- Receiving radical cystectomy (with lymph node dissection) or nephroureterectomy
- Histologically confirmed (surgical specimen) muscle-invasive urothelial carcinoma, and the major histological type should be transitional cell carcinoma
- Classification of tumor, node, and metastasis: pT2-4a N0-2 M0
- Absence of microscopic (ie, positive margin) or gross residual of the tumor (R0 resection) and absence of metastasis, confirmed by a negative computed tomography or magnetic resonance imaging scan of pelvis, abdomen, and chest within 4 weeks before enrollment
- Adequate hematologic and end-organ function

**Exclusion criteria**

- Receiving any approved anticancer treatment within 3 weeks before study enrollment
- Participation in another clinical trial with therapeutic intent within 28 days before enrollment
- Experiencing malignancies other than urothelial carcinoma within 2 years before study enrollment
- Conditions that contraindicate chemotherapy, such as renal impairment with creatinine clearance rate <50 mL/min, hearing impairment, and inadequate marrow function
- Anaphylactic or hypersensitivity reactions or other contraindication to cisplatin and gemcitabine
- Active or uncontrolled infections, including HIV, hepatitis B virus, hepatitis C virus, or tuberculosis
- Pregnancy or breastfeeding

### Sample Size Estimation

Given this is a pilot study intended as a preliminary exploration, the sample size calculation is based on other similar research and a rule of thumb rather than a formal power calculation [21]. As a pilot study, sufficient statistical power is not expected. The preliminary data from this pilot study will help us estimate the sample size in the following phase 3 trial with a scaled-up sample. On the basis of the limited sources of data, assuming a superiority margin of 26% points for the analysis of 1-year DFS in the GC versus SM arms (probability) [19], with a type-I error of 5% (95% confidence) and a randomization ratio of 1:1, a total sample of 20 patients (10 patients with ctDNA MRD-positive status and 10 patients with ctDNA MRD-negative status) will be deemed sufficient to investigate the acceptability, feasibility, and preliminary impact of the GC intervention. According to the medical records from the 2 hospitals, it is feasible for the 2 study sites to recruit 20 participants within the given timeline.

### Genetic Profiling

#### Sample Collection and Processing

Blood samples (10 mL×2 tubes per participant) will be collected in Streck tubes on the day of the operation. The fresh resected tumor tissue (≥50 mg) will be wiped up by gauze or filter paper immediately and then fixed in specimen bottles using 10% formalin (≥60% of the bottle) within 30 minutes. Samples will be stored at 6 to 37 °C for transport to the laboratory within 72 hours for further analysis.

#### Whole Exome Sequencing

A median of 500 ng of genomic DNA will be used for the WES workflow for the primary tumor tissue and white blood cells, with an average deduplicated on-target read depth of 300× for tumor DNA and 100× for associated matched germline samples. Targeted exome capture, including tumor mutation burden, microsatellite instability, and human leukocyte antigens, will be performed using a capture probe set with high specificity. The sequencing will be implemented by the brPROPHET (Burning Rock Biotech) service [15,22].

#### Somatic Variant Calling and Bespoke ctDNA Assay Design

Patient-specific somatic variants from the paired primary tumors and matched white blood cell DNA WES profiles (as mentioned previously) will be analyzed for quality control and sample concordance and then processed via a bioinformatics pipeline to identify somatic single-nucleotide variants (SNVs). A total of 50 highly ranked compatible amplicons (whose variant allele frequency is ≥5%) will be selected for the patient-specific panel. Plasma cell-free DNA will be extracted using the QIAamp Circulating Nucleic Acid Kit (Qiagen). No less than 20 ng will be used as the input for library preparation. Finally, a multiplex targeted PCR will be performed to detect ctDNA MRD, followed by amplicon-based sequencing at an average NGS read depth of up to 100,000×, with a target limit of detection as low as 0.004%. A personalized, tumor-informed ctDNA-based NGS assay will be implemented using the brPROPHET service [15,22].

The paired tumor and whole blood samples will be collected on day 0 for WES (operation day). The genetic signature profile of ctDNA via a 20-mL blood sample will be performed on day 28 (baseline before chemotherapy) and weeks 12, 24, 36, and 48 after surgery (follow-up at 4 time points).

### Randomization and Allocation

Patients will be stratified by their baseline ctDNA MRD results and randomly allocated in a 1:1 ratio to either a 4-cycle GC chemotherapy arm or an SM arm using computer-generated random numbers for simple randomization:

- ctDNA MRD-positive: GC arm (n=5) versus SM arm (n=5)
- ctDNA MRD-negative: GC arm (n=5) versus SM arm (n=5)

A sealed envelope method will be used for group allocation. Specifically, the sequentially numbered envelope containing the allocation sequence will be opened by a research assistant, and the participant will be informed of the allocation.

Blinding is not applicable in this pilot study. However, the treatment group will be deidentified during the data analysis stage, and a collaborator from a third party with no knowledge of the randomization plan will help to review the data analysis.

### Interventions

#### GC Chemotherapy: Intervention Group

GC is the recommended first-line standard therapy for MIUC. Patients will be treated with 1000-mg/m^2^ intravenous gemcitabine on day 1 and day 8 plus 80-mg/m^2^ intravenous cisplatin on day 1 or day 2 every 3 weeks (1 cycle) for 4 cycles. A reduced dose of GC will be acceptable if the patient is not suitable for a full dose of GC. For instance, patients with a creatinine clearance rate of 50 to 60 mL/min will receive a 75% dosage of GC. A 3-cycle regimen will also be considered upon an oncologist’s evaluation.

#### SM: Control Group

As a standard of care in Hong Kong, regular follow-up will be provided, and deferred chemotherapy will be initiated until radiological progression is confirmed.

### Baseline Assessments

Sociodemographic information (eg, age, gender, employment status, educational level, and marital status); baseline quality of life (QoL); and baseline fear of cancer recurrence (FCR) will be collected using a structured questionnaire (Multimedia Appendix 3). The clinical information of each participant will be collected from the Clinical Management System using a case report form (Multimedia Appendix 4). A baseline radiological evaluation (computed tomography scan of the thorax, abdomen, and pelvis) will be implemented on day 28 to confirm the absence of radiological relapse in patients with ctDNA MRD-positive status.

### Outcomes and Assessments

The primary outcome is feasibility, which will be evaluated using 4 indicators: recruitment, retention, adherence, and completeness, with the definition and predetermined criteria shown in Table 1.

**Table 1:** Definition and criteria to assess the feasibility of the trial.

Indicator	Definition	Benchmark
Recruitment	Eligible patients agreeing to participate in the trial	≥20% recruitment response rate
Retention	Completion of the trial and assessments during the follow-up	≤20% loss to follow-up or withdrawal
Adherence	Adherence to the allocated treatment in the gemcitabine plus cisplatin arm	≥80% intervention adherence
Completeness	Completeness and quality of collected data	≤20% missing value rate

The secondary outcome is treatment-related adverse events (AEs). AEs will be reported in accordance with the Common Terminology Criteria for Adverse Events (version 5.0) [23].

Exploratory outcomes include radiographic DFS, cancer-specific survival, OS, ctDNA clearance in patients with ctDNA MRD-positive status, QoL, FCR, and cost-effectiveness. For radiographic DFS, the radiographic assessment is based on the Response Evaluation Criteria in Solid Tumours (version 1.1), refer to the Follow-Up Strategy section. The radiographic DFS time is calculated from the date of randomized allocation to the date of confirmation of cancer recurrence (event time), death, or dropout, or the last date when the patient is still free of disease (censoring time), whichever occurs earlier. The cancer-specific survival time is calculated from the date of death from urothelial cancer. The OS time is calculated from the date of randomized allocation to the date of death. ctDNA clearance is defined as the change from ctDNA positive to ctDNA-negative status after postsurgical treatment. QoL will be assessed by the Functional Assessment of Cancer Therapy—Bladder [24], which is a 39-item questionnaire comprising physical, social, emotional, and functional well-being, and a bladder cancer subscale, with a higher total score indicating a higher QoL level. FCR will be estimated using the Fear of Cancer Recurrence Inventory–Short Form [25], which is a 9-item questionnaire assessing the presence and severity of intrusive thoughts related to FCR, with a higher score reflecting a higher FCR level. Cost-effectiveness will be assessed by the incremental cost-effectiveness ratio, which is calculated by dividing the difference in costs between GC and SM treatment by the difference in effects, where costs are estimated based on a health care perspective and effects consist of radiographic DFS, time to recurrence, and OS. The assessment schedule of the entire study is listed in Multimedia Appendix 5.

### Follow-Up Strategy

**Figure 1:**
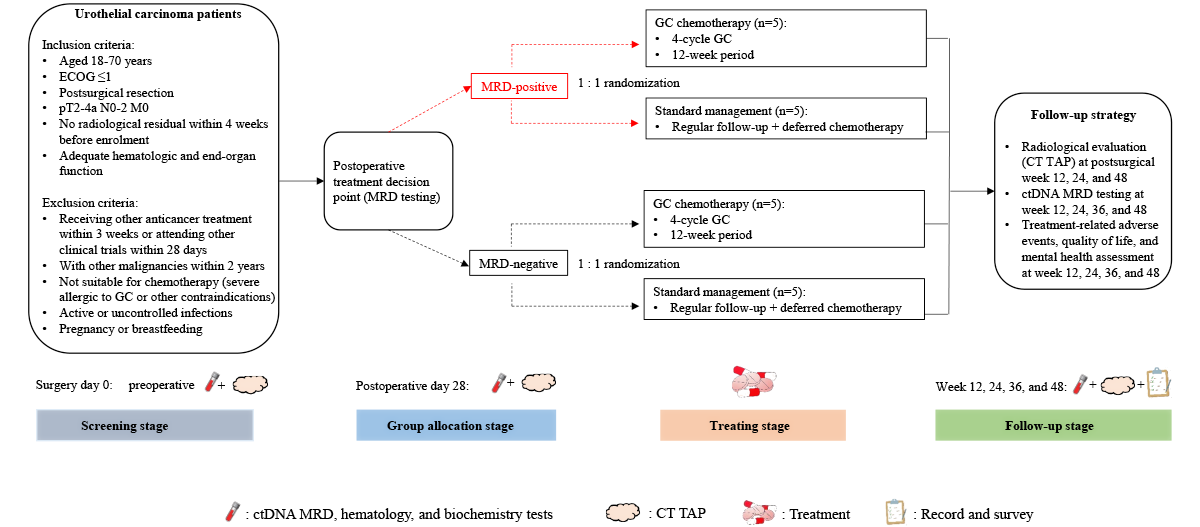
Flowchart of the proposed pilot randomized controlled trial. ctDNA: circulating tumor DNA; CT TAP: computed tomography scan of thorax, abdomen, and pelvis; ECOG: Eastern Cooperative Oncology Group; GC: gemcitabine plus cisplatin; MRD: molecular residual disease.

Patients will be followed up by radiological evaluation (computed tomography scan of the thorax, abdomen, and pelvis) at weeks 12, 24, and 48 after surgery. The genetic signature profiles of ctDNA, treatment-related AEs, QoL, and FCR will be assessed at follow-up at weeks 12, 24, 36, and 48 after surgery. A flowchart of the study design is presented in Figure 1. A long-term follow-up of every 6 months will continue to be implemented for each participant after the end of this study.

### Trial Oversight and Discontinuation or Withdrawal Criteria

#### Trial Oversight

All research personnel will receive training and comply with the standard operating procedures and the guidelines of the Declaration of Helsinki, the International Council for Harmonisation Good Clinical Practice, and the HKU Health System framework. The study procedure and safety evaluation will be monitored by a research assistant. Participants will be reminded of the scheduled follow-up by a telephone call. Any problems will be addressed by the project coordinator during the course of the study.

#### Treatment Discontinuation

Treatment discontinuation will be determined after sufficient evaluation by the investigator if participants have any of the following conditions: (1) disease progression during adjuvant chemotherapy; (2) symptom deterioration caused by disease progression, such as uncontrollable pain; (3) unexpected severe AEs from the intervention; (4) receiving other anticancer regimens; and (5) getting pregnant. Discontinuation of the study treatment alone does not constitute a withdrawal from the study.

#### Participation Withdrawal

Participants can withdraw from any study period at any time without giving reasons and experiencing any disadvantages. Their personal data, along with biospecimens, will be destroyed upon request.

#### Loss to Follow-Up

Participants will be deemed lost to follow-up if they continuously fail to attend the scheduled visits or continue to be uncontactable or unreachable during the study period. The detailed remedial actions for loss to follow-up are described in the “Contingency Plans” section.

### Patient and Public Involvement

Given that GC chemotherapy is the recommended first-line standard therapy for MIUC, patients were not involved in the recruitment and intervention process. However, patients can still benefit from the dynamic MRD monitoring, and the results showing the ctDNA-based MRD trajectory will be sent to every participant at the completion of participation. The side effects of the GC chemotherapy are explained to all participants, who are encouraged to report any symptoms for the duration of the study.

### Data Processing and Management

#### Clinical and Survey Data Processing

The investigator will input the baseline, treatment, follow-up, and outcome data into Microsoft Excel. Survey data entry into Microsoft Excel with preprogrammed check rules will be conducted independently by 2 personnel.

#### ctDNA Data Transfer and Processing

Patient-specific somatic variants from the WES profile will be analyzed for quality control and sample concordance and then processed via a bioinformatics pipeline to identify somatic SNVs. Bespoke PCR amplicons will be designed based on a prioritized list of the detected SNVs. After plasma cell-free DNA extraction and library preparation, a multiplex targeted PCR will be performed, followed by amplicon-based sequencing. An observation of at least 2 variants within each plasma sample will be defined as ctDNA positive [13,19].

#### Data Storage

The data storage and processing will comply with the university security code. All clinical and survey data will be kept fully confidential and will be inputted on a dedicated computer with an internet connection and updated antivirus software. The process and reagent or equipment information of the sample processing and genetic profiling will be captured electronically and uploaded to a database with built-in integrity checks. All the data will be backed up onto the team server. Files in each follow-up stage will be backed up on an external hard drive for an additional safety purpose

### Statistical Analysis

#### Descriptive Statistics

Numbers with proportions or means with SDs will be calculated to describe the characteristics among the 4 groups (MRD-positive with GC, MRD-positive with SM, MRD-negative with GC, and MRD-negative with SM), including baseline demographics, presurgical examinations, postsurgical examinations, and outcome assessments.

#### Feasibility Evaluation

Benchmarks for feasibility outcomes are set as (1) ≥20% recruitment response rate, (2) ≤20% loss to follow-up or withdrawal, (3) ≥80% intervention adherence, and (4) ≤20% missing value rate. Each benchmark will be assigned one score, and a total score of 4, 2 to 3, and 0 to 1 will be deemed high, medium, and low feasibility, respectively.

Safety evaluation will be presented as numbers and proportions of the AEs. ANOVA and the Kruskal-Wallis test will be used for continuous outcome variables, whereas the chi-square test and the Fisher exact test will be used for categorical outcome variables.

#### Efficacy Analysis

Hazard ratios and risk difference will be calculated to compare the treatment effect of the GC arm against the SM arm on survival within each MRD group. As this is a pilot study, we will mainly focus on the magnitude and direction of the preliminary effect sizes, and there will be no anticipation of finding statistically significant differences between the groups.

All process indicators will be presented in accordance with the CONSORT (Consolidated Standards of Reporting Trials) guidelines. Data processing and analyses will mainly be based on the following software: Microsoft Excel, Stata (StataCorp LLC), and R statistics (R Foundation for Statistical Computing).

### Contingency Plans

Detailed training will be provided to all research staff, all procedure will be conducted based on the protocol, and all issues will be negotiated and addressed by the same project coordinator in two study sites to ensure site activation in time.

All eligible participants will be documented at the enrolment stage. In case of participant withdrawal, an additional consenting and randomized allocation will be performed from the waiting list as appropriate.

At recruitment stage, contact persons, email address, and mail address should be documented in participants’ medical records. If one misses a scheduled visit but can be contactable, a rescheduled visit should be arranged as soon as possible. If one continues to be unreachable during the follow-up, every effort should be made to regain contact with him or her, including telephone call to him or her and his or her contact persons, a reminder sent to his or her email address, a certified letter posted to his or her mailing address, and home visit as appropriate.

## Results

The project was funded in February 2024. Patient recruitment started on May 2, 2024 (first patient’s first visit). Recruitment and data collection for the trial are ongoing. Data analysis will be performed in mid-2025 and the results of this study are expected to be published in late 2025.

## Discussion

### Anticipated Findings

To the best of our knowledge, this pilot RCT is the first to frame a contextual ctDNA-guided approach for postoperative adjuvant therapy in patients with urothelial carcinoma. The feasibility and preliminary understanding of effect sizes will be used to inform sample size and refine procedures for a further scale-up and definitive RCT for efficacy and cost-effectiveness estimates.

In this pilot study, the results of the feasibility and safety assessment will help refine the future large-scale trial in terms of inclusion criteria, recruitment procedure, implementation process, and discontinuation and withdrawal criteria. In addition, the magnitude and direction of the preliminary effect sizes calculated in this study will allow for the estimation of the sample size for a larger RCT.

The long-term benefits of this study will mainly stem from the results of the subsequent phase 3 clinical trial. For instance, patient care will be significantly improved; patients with cancer and detected MRD will be timely treated, while those without MRD will be spared from overtreatment and unnecessary toxicity. Hopefully, clinical practice will be improved to a more personalized pattern based on genomics and precision medicine. The future translation and implementation of the research findings and the new technology may also benefit the commercialization of the test and the relevant health care industry.

### Limitations

The main limitation of this study is the pilot design with a small sample size, resulting in insufficient statistical power to detect significant differences between groups. Nevertheless, the effect size (ie, hazard ratio and risk difference) will be used to inform a definitive scale-up trial. As this trial is performed within Hong Kong, generalizability is another limitation, and the results of the study may not reflect outcomes representative of the broader population. Finally, given the nature of the intervention, blinding of the participants and the interventionists is impractical.

### Conclusions

This study will provide preliminary evidence of personalized management for postoperative urothelial carcinomas, including personalized treatment and early detection of disease progression. This may bring significant benefits to patients and reduce the burden from the potential overtreatment or side effects in the health care system and society.

## Appendix

Multimedia Appendix 1 Genetic characteristics of the 3 samples undergoing whole exome sequencing.

Multimedia Appendix 2 Circulating tumor DNA surveillance at baseline, treatment, and follow-up.

Multimedia Appendix 3 Questionnaire.

Multimedia Appendix 4 Case report form.

Multimedia Appendix 5 Assessment schedule of the study.

Multimedia Appendix 6 Peer-review report from the Health and Medical Research Fund Research Council Grant Review Board (Hong Kong).

## Abbreviations

AE: adverse event
CONSORT: Consolidated Standards of Reporting Trials
ctDNA: circulating tumor DNA
DFS: disease-free survival
FCR: fear of cancer recurrence
GC: gemcitabine plus cisplatin
MIUC: muscle-invasive urothelial carcinoma
MRD: molecular residual disease
NGS: next-generation sequencing
OS: overall survival
PCR: polymerase chain reaction
QoL: quality of life
RCT: randomized controlled trial
SM: standard management
SNV: single-nucleotide variant
WES: whole exome sequencing

## Data Availability

The trial dataset is not available publicly, and any requests for access should be sent to the corresponding author.
