# Supplementary material for: Surveillance of the Genetic Signature in Circulating Tumor DNA for Guiding Adjuvant Chemotherapy in Urothelial Carcinoma: Protocol for a Pilot Randomized Controlled Trial
Source: JMIR Res Protoc. 2025 Aug 26;14:e72597. doi: 10.2196/72597 (PMC12421199; doi:10.2196/72597)
Supplement: Multimedia Appendix 1 [file resprot_v14i1e72597_app1.pdf]

Appendix 1: Genetic characteristics of the three samples undergoing whole exome sequencing

	Sample 1	Sample 2	Sample 3
**Cancer type**	Bladder cancer	Bladder cancer	Bladder cancer
**Sample type**	FFPE	FFT	FFT
**No. genetic signatures**	98	481	120
**Mutation types with numbers**			
Missense mutation	85	260	93
Nonsense mutation	7	21	4
Alternative splicing	3	6	3
Frameshift mutation	1	4	7
Splice site mutation	0	8	4
Initiation codon deletion	0	1	0
In-frame deletion	0	1	2
Rearrangement	0	0	3

FFPE, Formalin-Fixed Paraffin-Embedded; FFT, Fresh Frozen Tissue
