# Supplementary material for: Surveillance of the Genetic Signature in Circulating Tumor DNA for Guiding Adjuvant Chemotherapy in Urothelial Carcinoma: Protocol for a Pilot Randomized Controlled Trial
Source: JMIR Res Protoc. 2025 Aug 26;14:e72597. doi: 10.2196/72597 (PMC12421199; doi:10.2196/72597)

Appendix 2: ctDNA surveillance at baseline, treatment, and follow-up (NAT, neoadjuvant therapy; MTM, mean tumor molecules)

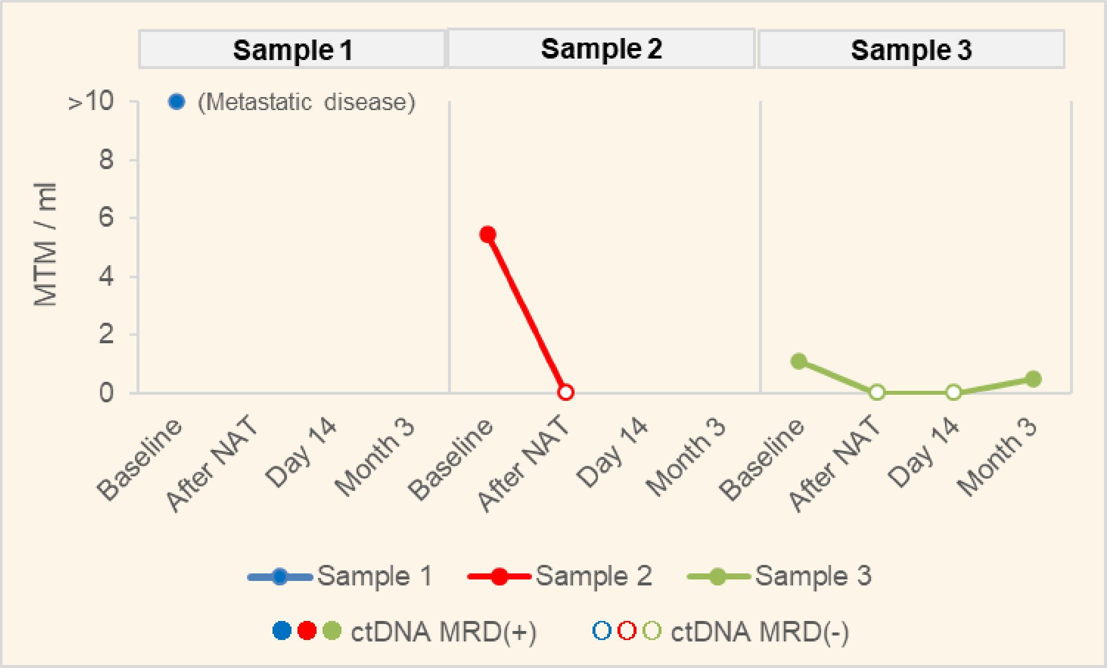

Supplement: Multimedia Appendix 2 [file resprot_v14i1e72597_app2.pdf]
