# Supplementary material for: Surveillance of the Genetic Signature in Circulating Tumor DNA for Guiding Adjuvant Chemotherapy in Urothelial Carcinoma: Protocol for a Pilot Randomized Controlled Trial
Source: JMIR Res Protoc. 2025 Aug 26;14:e72597. doi: 10.2196/72597 (PMC12421199; doi:10.2196/72597)
Supplement: Multimedia Appendix 3 [file resprot_v14i1e72597_app3.pdf]

Appendix 3: Questionnaire

Surveillance of the Genetic Signature in Circulating Tumor DNA for Guiding Adjuvant Chemotherapy in Urothelial Carcinoma: A Pilot Randomized Controlled Trial

Good morning / afternoon / evening, my name is ___________. I am an interviewer of _____________. We are conducting a survey on behalf of the Department of Surgery, University of Hong Kong. The survey aims to evaluate the quality of life, fear of cancer recurrence and your lifestyle habits before and after postsurgical treatment. Please be assured that the information provided by you will be kept strictly confidential. The interview will take about 15 minutes for each person. Do you have that time available now? Thanks for your co-operation.

Interviewer ID:

Section A: Background Information

A1 Patient ID:

A2 Sex:

(01) Male

(02) Female

A3 Date of Birth (DD/MM/YY):

A4 What is the highest level of school education you ever received?

(01) Primary or below

(02) Secondary (middle / high school)

(03) Technical school / college

(04) University and above

(98) Don’t know

(99) Refuse to answer

A5 What is your current occupation?

(01) Agriculture & related workers

(02) Factory worker

(03) Administrator / manager

(04) Professional / technical

(05) Sales & service workers

(06) Retired

(07) House wife / husband

(08) Self-employed

(09) Unemployed

(10) Other or not stated

(98) Don’t know
(99) Refuse to answer

A6 How many people living together in the household? ______ person

A7 What is your current marital status?
(01) Never married
(02) Married
(03) Widowed
(04) Divorced / separated
(98) Don’t know
(99) Refuse to answer

A8 Including all your income sources and mandatory provident fund contribution, what is your average monthly household income in total? (HK$ / month)?
(01) $<10,000
(02) $10,000 - $19,999
(03) $20,000 - $39,999
(04) $≥40,000
(98) Don’t know
(99) Refuse to answer

Section B: Quality of Life

Below is a list of statements that other people with your illness have said are important. Please mark one number per line to indicate your response as it applies to the ***past 7 days***.

0	1	2	3	4
Not at all	A little bit	Somewhat	Quite a bit	Very much

B1 Physical Well-being:		0	1	2	3	4
B1.1	I have a lack of energy.	○	○	○	○	○
B1.2	I have nausea.	○	○	○	○	○
B1.3	Because of my physical condition, I have trouble meeting the needs of my family.	○	○	○	○	○
B1.4	I have pain.	○	○	○	○	○
B1.5	I am bothered by side effects of treatment.	○	○	○	○	○
B1.6	I feel ill.	○	○	○	○	○
B1.7	I am forced to spend time in bed.	○	○	○	○	○
B2 Social/Family Well-being:		0	1	2	3	4
B2.1	I feel close to my friends.	○	○	○	○	○
B2.2	I get emotional support from my family.	○	○	○	○	○

B2.3	I get support from my friends.	○	○	○	○	○
B2.4	My family has accepted my illness.	○	○	○	○	○
B2.5	I am satisfied with family communication about my illness.	○	○	○	○	○
B2.6	I feel close to my partner (or the person who is my main support).	○	○	○	○	○
Regardless of your current level of sexual activity, please answer the following question. If you prefer not to answer it, please mark this box□ and go to the next section.						
B2.7	I am satisfied with my sex life.	○	○	○	○	○
B3 Emotional well-being:		0	1	2	3	4
B3.1	I feel sad.	○	○	○	○	○
B3.2	I am satisfied with how I am coping with my illness.	○	○	○	○	○
B3.3	I am losing hope in the fight against my illness.	○	○	○	○	○
B3.4	I feel nervous.	○	○	○	○	○
B3.5	I worry about dying.	○	○	○	○	○
B3.6	I worry that my condition will get worse.	○	○	○	○	○
B4 Functional well-being:		0	1	2	3	4
B4.1	I am able to work (include work at home).	○	○	○	○	○
B4.2	My work (include work at home) is fulfilling.	○	○	○	○	○
B4.3	I am able to enjoy life.	○	○	○	○	○
B4.4	I have accepted my illness.	○	○	○	○	○
B4.5	I am sleeping well.	○	○	○	○	○
B4.6	I am enjoying the things I usually do for fun.	○	○	○	○	○
B4.7	I am content with the quality of my life right now.	○	○	○	○	○
B5 Additional concerns:		0	1	2	3	4
B5.1	I have trouble controlling my urine.	○	○	○	○	○
B5.2	I am losing weight.	○	○	○	○	○
B5.3	I have control of my bowels.	○	○	○	○	○
B5.4	I urinate more frequently than usual.	○	○	○	○	○
B5.5	I have diarrhea (diarrhoea).	○	○	○	○	○
B5.6	I have a good appetite.	○	○	○	○	○
B5.7	I like the appearance of my body.	○	○	○	○	○
B5.8	It burns when I urinate.	○	○	○	○	○
B5.9	I am interested in sex.	○	○	○	○	○
B5.10	(For men only) I am able to have and maintain	○	○	○	○	○

	an erection					
Do you have an ostomy appliance? □*Yes* □*No (→ C)*						
If yes, answer the following two items:						
B5.11	I am embarrassed by my ostomy appliance.	○	○	○	○	○
B5.12	Caring for my ostomy appliance is difficult.	○	○	○	○	○

Section C: Fear of Cancer Recurrence

Most people who have been diagnosed with cancer are worried, to varying degrees, that there might be a recurrence of the cancer. ***By recurrence, we mean the possibility that the cancer could return or progress in the same place or in another part of the body***. This section aims to better understand the experience of worries about cancer recurrence. Please read each statement and indicate to what degree it applied to you ***DURING THE PAST MONTH*** by circling the appropriate number.

0	1	2	3	4
Not at all	A little	Somewhat	A lot	A great deal

		0	1	2	3	4
C1	I am worried or anxious about the possibility of cancer recurrence.	○	○	○	○	○
C2	I am afraid of cancer recurrence.	○	○	○	○	○
C3	I believe it is normal to be worried or anxious about the possibility of cancer recurrence.	○	○	○	○	○
C4	When I think about the possibility of cancer recurrence, this triggers other unpleasant thoughts or images (such as death, suffering, the consequences for my family).	○	○	○	○	○
C5	I believe that I am cured and that the cancer will not come back.	○	○	○	○	○
C6	In your opinion, are you at risk of having a cancer recurrence?	○	○	○	○	○

C7 How often do you think about the possibility of cancer recurrence?

0	○	Never
1	○	A few times a month
2	○	A few times a week
3	○	A few times a day
4	○	Several times a day

C8 How much time <u>per day</u> do you spend thinking about the possibility of

cancer recurrence?

0	○	I don’t think about it
1	○	A few seconds
2	○	A few minutes
3	○	A few hours
4	○	Several hours

C9 How long have you been thinking about the possibility of cancer recurrence?

0	○	I don’t think about it
1	○	A few weeks
2	○	A few months
3	○	A few years
4	○	Several years

Section D: Lifestyle habits

D1 Have you ever / do you current smoke cigarettes?

(00) Never

(01) Ever, have quitted already

(03) Current smoker

D2 Have you ever / do you currently drink alcohol?

(00) Never

(01) Ever, have quitted already

(03) Current drinker

D3 In the past week, how often (day/week) have you consumed the following foods?

	Food group	***0***	***1***	***2***	***3***	***4***	***5***	***6***	***7***
D3.1	Cereal (rice, wheat, corn, etc.)	○	○	○	○	○	○	○	○
D3.2	Fresh fruit	○	○	○	○	○	○	○	○
D3.3	Dried fruit	○	○	○	○	○	○	○	○
D3.4	Raw vegetables	○	○	○	○	○	○	○	○
D3.5	Cooked vegetables	○	○	○	○	○	○	○	○
D3.6	Bean curd or other soya products (soy milk, soy yogurt)	○	○	○	○	○	○	○	○
D3.7	Cheese, yogurt and dairy products	○	○	○	○	○	○	○	○
D3.8	Fish (excluding salted fish)	○	○	○	○	○	○	○	○
D3.9	Seafood (excluding fish)	○	○	○	○	○	○	○	○

D3.10	Other meat (e.g. beef, pork, poultry)	○	○	○	○	○	○	○	○
D3.11	Eggs	○	○	○	○	○	○	○	○
D3.12	Ice cream	○	○	○	○	○	○	○	○
D3.13	Preserved / processed meat	○	○	○	○	○	○	○	○
D3.14	Preserved / processed vegetable	○	○	○	○	○	○	○	○

D4 How many glasses of water do you drink each DAY: _______ glasses

D5 Physical activity:

We are interested in finding out about the kinds of physical activities that people do as part of their everyday lives.

The questions will ask you about the time you spent being physically active in the last 7 days. Please answer each question even if you do not consider yourself to be an active person.

Please think about the activities you do at work, as part of your house and yard work, to get from place to place, and in your spare time for recreation, exercise, or sport.

Think about all the vigorous activities that you did in the last 7 days. Vigorous physical activities refer to activities that take hard physical effort and make you breathe much harder than normal.

Think only about those physical activities that you did for at least 10 minutes at a time.

D5.1 During the last 7 days, on how many days did you do vigorous physical activities like heavy lifting, digging, aerobics, or fast bicycling?

(00) No vigorous physical activities → ***D5.3***

(01) _____days per week

D5.2 How much time did you usually spend doing vigorous physical activities on one of those days? _____hours _____minutes per day

Think about all the moderate activities that you did in the last 7 days. Moderate activities refer to activities that take moderate physical effort and make you breathe somewhat harder than normal.

Think only about those physical activities that you did for at least 10 minutes at a time.

D5.3 During the last 7 days, on how many days did you do moderate physical activities like carrying light loads, bicycling at a regular pace, or doubles tennis? Do not include walking.

(00) No moderate physical activities → ***D5.5***

(01) _____days per week

D5.4 How much time did you usually spend doing mmoderate physical activities on one of those days? _____hours _____minutes per day

Think about the time you spent walking in the last 7 days. This includes at work and at home, walking to travel from place to place, and any other walking that you have done

solely for recreation, sport, exercise, or leisure.

D5.5 During the last 7 days, on how many days did you walk for at least 10 minutes at a time?

(00) No walking → ***D5.7***

(01) _____days per week

D5.6 How much time did you usually spend walking on one of those days?

_____hours _____minutes per day

The last question is about the time you spent sitting on weekdays during the last 7 days. Include time spent at work, at home, while doing course work and during leisure time. This may include time spent sitting at a desk, visiting friends, reading, or sitting or lying down to watch television.

D5.7 During the last 7 days, how much time did you spend sitting on a typical day?

_____ hours _____minutes per day

D6 During the past month, did you have any of the following for ≥3 days each week?

	Yes	No
D6.1 Taking >30 minutes to fall asleep after going to bed or waking up in the middle of the night	○	○
D6.2 Waking up early and not being able to go back to sleep	○	○
D6.3 Needing to take medicine (including herbal or sleeping pills) at least once a week to help sleep	○	○
D6.4 Having difficulty staying alert while at work, eating or meeting people during daytime	○	○

D7 How many hours do you typically sleep per day (include naps): __hours

~ END ~
