# Supplementary material for: Surveillance of the Genetic Signature in Circulating Tumor DNA for Guiding Adjuvant Chemotherapy in Urothelial Carcinoma: Protocol for a Pilot Randomized Controlled Trial
Source: JMIR Res Protoc. 2025 Aug 26;14:e72597. doi: 10.2196/72597 (PMC12421199; doi:10.2196/72597)
Supplement: Multimedia Appendix 4 [file resprot_v14i1e72597_app4.pdf]

Appendix 4: Case Report Form

CASE REPORT FORM

PROJECT TITLE: Surveillance of the Genetic Signature in Circulating Tumor DNA for Guiding Adjuvant Chemotherapy in Urothelial Carcinoma: A Pilot Randomized Controlled Trial

Patient Number: | | | | |

Study Center: | | |

General Instructions for Completion of the Case Report Forms (CRF)

Completion of CRFs

- A CRF must be completed for each study patient who is successfully enrolled.
- For reasons of confidentiality, the name and initials of the study patient should **not** appear on the CRF.

General

- Please print all entries in BLOCK CAPITAL LETTERS using a **black** ballpoint pen.
- All text and explanatory comments should be brief.
- Answer every question explicitly; do not use ditto marks.
- Do not leave any question unanswered. If the answer to a question is unknown, write "**NK**" (Not Known). If a requested test has not been done, write "**ND**" (Not Done). If a question is not applicable, write "**NA**" (Not Applicable).
- Where a choice is requested, **cross (X)** the appropriate response.

Dates and Times

- All date entries must appear in the format DD-MMM-YYYY e.g., 01-Jan-2023. The month abbreviations are as follows:

January	=	Jan	May	=	May	September	=	Sep
February	=	Feb	June	=	Jun	October	=	Oct
March	=	Mar	July	=	Jul	November	=	Nov
April	=	Apr	August	=	Aug	December	=	Dec

  In the absence of a precise date for an event or therapy that precedes the participant's inclusion into the study, a partial date may be recorded by recording "NK" in the fields that are unknown e.g., where the day and month are not clear, the following may be entered into the CRF:

N	K	N	K		2	0	2	3
DD		MMM			YYYY			

- All time entries must appear in **24-hour format** e.g. 13:00., Entries representing midnight should be recorded as 00:00 with the date of the new day that is starting at that time.

Correction of Errors

- **Do not** overwrite erroneous entries or use correction fluid or erasers.
- Draw a straight line through the entire erroneous entry without obliterating it.
- Clearly enter the correct value next to the original (erroneous) entry.
- Date and initial the correction.

PARTICIPANT INFORMATION

**Patient Number**	☐☐☐☐	
**Date of Informed Consent**	D D M M M Y Y Y Y	
**Inclusion criteria** (Did the subject meet the following criteria requirements for inclusion?)	Yes 1.	No 2.
(01) aged 18-75 years old	☐	☐
(02) histologically confirmed (surgical specimen) muscle invasive bladder cancer, urothelial carcinoma in renal pelvis or ureter (pT2-pT4a N0-2M0), and the majority histological type should be transitional cell carcinoma	☐	☐
(03) receiving radical cystectomy or nephroureterectomy with or without lymph node dissection	☐	☐
(04) no post-operational radiological residual of the tumor	☐	☐
(05) with a ≤1 score of the Eastern Cooperative Oncology Group (ECOG) Performance Status	☐	☐
**Exclusion criteria** (Does the subject have any of the following that should be excluded from the study)	Yes 1.	No 2.
(01) receiving any approved anti-cancer treatment including steroid or participating in other clinical trials within 28 days prior to the screening	☐	☐
(02) patients with other conditions that are not eligible to the chemotherapy even with the reduced doses, such as end stage renal failure, severe allergic to platinum, cisplatin and gemcitabine, and other contraindication	☐	☐
(03) other untreated or uncontrollable diseases and active infections	☐	☐
Date of Birth	D D M M M Y Y Y Y	Or estimated age: _______
Gender	☐1 Male ☐2 Female	
Pregnant (only for female)	☐1. Yes	☐2. No ☐9. Unknown
If pregnant, Estimated Gestational Age ___________weeks		
Date of Enrolment	D D M M M Y Y Y Y	

PRESURGICAL SYMPTOMS			
Fever (in last 24 hours)	☐1. Yes	☐2. No	Duration: _______ days
Dizziness	☐1. Yes	☐2. No	Duration: _______ days
Headache	☐1. Yes	☐2. No	Duration: _______ days
Nausea	☐1. Yes	☐2. No	Duration: _______ days
Anorexia	☐1. Yes	☐2. No	Duration: _______ days
Vomiting	☐1. Yes	☐2. No	Duration: _______ days
Diarrhoea	☐1. Yes	☐2. No	Duration: _______ days
Abdominal pain	☐1. Yes	☐2. No	Duration: _______ days
Itching	☐1. Yes	☐2. No	Duration: _______ days
Skin rash	☐1. Yes	☐2. No	Duration: _______ days
Urticaria	☐1. Yes	☐2. No	Duration: _______ days
Joint pain	☐1. Yes	☐2. No	Duration: _______ days
Muscle pain	☐1. Yes	☐2. No	Duration: _______ days
Palpitations	☐1. Yes	☐2. No	Duration: _______ days
Dyspnoea	☐1. Yes	☐2. No	Duration: _______ days
Hearing problem	☐1. Yes	☐2. No	Duration: _______ days
Confusion	☐1. Yes	☐2. No	Duration: _______ days
Visual blurring	☐1. Yes	☐2. No	Duration: _______ days
Fatigue	☐1. Yes	☐2. No	Duration: _______ days
Other symptom:	____________________		Duration: _______ days
Other symptom:	____________________		Duration: _______ days
Other symptom:	____________________		Duration: _______ days

MEDICATION HISTORY (within the last 7 days)

- Make multiple copies of this page if required

**Medication Name** (write NK if unknown)	**Start Date**	**Stop Date**
______________________	D D M M M Y Y Y Y	D D M M M Y Y Y Y
	OR ☐1 Unknown	*OR* ☐1 Ongoing
______________________	D D M M M Y Y Y Y	D D M M M Y Y Y Y
	OR ☐1 Unknown	*OR* ☐1 Ongoing
______________________	D D M M M Y Y Y Y	D D M M M Y Y Y Y
	OR ☐1 Unknown	*OR* ☐1 Ongoing
______________________	D D M M M Y Y Y Y	D D M M M Y Y Y Y
	OR ☐1 Unknown	*OR* ☐1 Ongoing
______________________	D D M M M Y Y Y Y	D D M M M Y Y Y Y
	OR ☐1 Unknown	*OR* ☐1 Ongoing
______________________	D D M M M Y Y Y Y	D D M M M Y Y Y Y
	OR ☐1 Unknown	*OR* ☐1 Ongoing
______________________	D D M M M Y Y Y Y	D D M M M Y Y Y Y
	OR ☐1 Unknown	*OR* ☐1 Ongoing
______________________	D D M M M Y Y Y Y	D D M M M Y Y Y Y
	OR ☐1 Unknown	*OR* ☐1 Ongoing
______________________	D D M M M Y Y Y Y	D D M M M Y Y Y Y
	OR ☐1 Unknown	*OR* ☐1 Ongoing
______________________	D D M M M Y Y Y Y	D D M M M Y Y Y Y
	OR ☐1 Unknown	*OR* ☐1 Ongoing
______________________	D D M M M Y Y Y Y	D D M M M Y Y Y Y
	OR ☐1 Unknown	*OR* ☐1 Ongoing
______________________	D D M M M Y Y Y Y	D D M M M Y Y Y Y
	OR ☐1 Unknown	*OR* ☐1 Ongoing
______________________	D D M M M Y Y Y Y	D D M M M Y Y Y Y
	OR ☐1 Unknown	*OR* ☐1 Ongoing

SIGNIFICANT MEDICAL HISTORY (within the past 5 years)

- Make multiple copies of this page if required

Does the patient have a history of any background/concomitant conditions/symptoms according to the following schedule? ☐[1] Yes ☐[2] No

If **Yes**, detail in the table below and reference the ICD10 system code http://apps.who.int/classifications/apps/icd/icd10online/

Code	Title	Code	Title
1	Certain infectious and parasitic diseases	12	Diseases of the skin and subcutaneous tissue
2	Neoplasms	13	Diseases of the musculoskeletal system and connective tissue
3	Diseases of the blood and blood-forming organs and certain disorders involving the immune mechanism	14	Diseases of the genitourinary system
4	Endocrine, nutritional and metabolic diseases	15	Pregnancy, childbirth and the puerperium
5	Mental and behavioural disorders	16	Certain conditions originating in the perinatal period
6	Diseases of the nervous system	17	Congenital malformations, deformations and chromosomal abnormalities
7	Diseases of the eye and adnexa	18	Symptoms, signs and abnormal clinical and laboratory findings, not elsewhere classified
8	Diseases of the ear and mastoid process	19	Injury, poisoning and certain other consequences of external causes
9	Diseases of the circulatory system	20	External causes of morbidity and mortality
10	Diseases of the respiratory system	21	Factors influencing health status and contact with health services
11	Diseases of the digestive system	22	Codes for special purposes

SIGNIFICANT MEDICAL HISTORY (within the past 5 years)

Code	Condition/Symptom	Onset Date	Stop Date
		D D M M M Y Y Y Y<br>*OR* ☐[1] Unknown	D D M M M Y Y Y Y<br>*OR* ☐[1] Ongoing
		D D M M M Y Y Y Y<br>*OR* ☐[1] Unknown	D D M M M Y Y Y Y<br>*OR* ☐[1] Ongoing
		D D M M M Y Y Y Y<br>*OR* ☐[1] Unknown	D D M M M Y Y Y Y<br>*OR* ☐[1] Ongoing
		D D M M M Y Y Y Y<br>*OR* ☐[1] Unknown	D D M M M Y Y Y Y<br>*OR* ☐[1] Ongoing
		D D M M M Y Y Y Y<br>*OR* ☐[1] Unknown	D D M M M Y Y Y Y<br>*OR* ☐[1] Ongoing

PRESURGICAL PHYSICAL EXAMINATION

Weight	____._ kg	Height	____._ cm

Temperature	__._ ℃	Method of Recording: Axillary ☐1	Tympanic ☐2	Rectal ☐3	Oral ☐4	Heart rate	___ bpm

Respiratory rate	__ bpm	Blood pressure	___ / ___ mmHg

Hepatomegaly	☐1. Yes	☐2. No	If yes, size: __ cm
Splenomegaly	☐1. Yes	☐2. No	If yes, size: __ cm

	**Normal**	**Abnormal**	**Specify if abnormal**
Central Nervous System	☐1.	☐2.	__________
Cardiovascular System	☐1.	☐2.	__________
Respiratory System	☐1.	☐2.	__________
Gastrointestinal System	☐1.	☐2.	__________
Skin	☐1.	☐2.	__________
Joints	☐1.	☐2.	__________

PRESURGICAL HAEMATOLOGY

Date<br>D D / M M M / Y Y Y Y	Time 24hr<br>H H : M M	Hb (g/dL)<br>___._	Hct (%)<br>___._	WBC (10^9/L)<br>___._
Neutrophils (%)<br>___._	Lymphocytes (%)<br>___._	Monocytes (%)<br>___._	Eosinophils (%)<br>___._	Platelets (10^9/L)<br>___._

CASE REPORT FORM

BASELINE

Participant Number	

PHYSICAL EXAMINATION - DAY 28

Date	D D M M M Y Y Y Y	Time	H H : M M
Weight	. kg	Height	. cm
Temperature	. °C	Method of Recording: Axillary ☐1 Tympanic ☐2 Rectal ☐3 Oral ☐4	Heart rate bpm
Respiratory rate	bpm	Blood pressure	/ m
Hepatomegaly	☐1. Yes ☐2. No	If yes, size:	cm
Splenomegaly	☐1. Yes ☐2. No	If yes, size:	cm

BASELINE HAEMATOLOGY – DAY 28

Date<br>D D M M M Y Y Y Y	Time 24hr<br>H H : M M	Hb (g/dL)<br>.	Hct (%)<br>.	WBC (10^9/L)<br>.
Neutrophils (%)<br>.	Lymphocytes (%)<br>.	Monocytes (%)<br>.	Eosinophils (%)<br>.	Platelets (10^9/L)<br>.

SYMPTOM CHECK – DAY 28

Date: D D M M M Y Y Y Y

Time 24hr: H H : M M

Symptom	Yes	No
Fever	☐	☐
Dizziness	☐	☐
Headache	☐	☐
Nausea	☐	☐
Anorexia	☐	☐
Vomiting	☐	☐
Diarrhoea	☐	☐
Abdominal pain	☐	☐
Itching	☐	☐
Skin rash	☐	☐
Urticaria	☐	☐
Joint pain	☐	☐
Muscle pain	☐	☐
Palpitations	☐	☐
Dyspnoea	☐	☐
Hearing problem	☐	☐
Confusion	☐	☐
Visual blurring	☐	☐
Fatigue	☐	☐

STUDY DRUG ADMINSTRATION – DAY 28

Study drug	Dose	Treatment observed?	Date of dose	Time of dose	Vomited?	Time of vomit	Retreatment?	Retreatment dose	Time of retreatment
___________	______	Yes ☐<br>No ☐	D D M M M Y Y Y Y	H H : M M	Yes ☐<br>No ☐	H H : M M	Yes ☐<br>No ☐	______	H H : M M
___________	______	Yes ☐<br>No ☐	D D M M M Y Y Y Y	H H : M M	Yes ☐<br>No ☐	H H : M M	Yes ☐<br>No ☐	______	H H : M M
___________	______	Yes ☐<br>No ☐	D D M M M Y Y Y Y	H H : M M	Yes ☐<br>No ☐	H H : M M	Yes ☐<br>No ☐	______	H H : M M
___________	______	Yes ☐<br>No ☐	D D M M M Y Y Y Y	H H : M M	Yes ☐<br>No ☐	H H : M M	Yes ☐<br>No ☐	______	H H : M M
___________	______	Yes ☐<br>No ☐	D D M M M Y Y Y Y	H H : M M	Yes ☐<br>No ☐	H H : M M	Yes ☐<br>No ☐	______	H H : M M
___________	______	Yes ☐<br>No ☐	D D M M M Y Y Y Y	H H : M M	Yes ☐<br>No ☐	H H : M M	Yes ☐<br>No ☐	______	H H : M M
___________	______	Yes ☐<br>No ☐	D D M M M Y Y Y Y	H H : M M	Yes ☐<br>No ☐	H H : M M	Yes ☐<br>No ☐	______	H H : M M
___________	______	Yes ☐<br>No ☐	D D M M M Y Y Y Y	H H : M M	Yes ☐<br>No ☐	H H : M M	Yes ☐<br>No ☐	______	H H : M M
___________	______	Yes ☐<br>No ☐	D D M M M Y Y Y Y	H H : M M	Yes ☐<br>No ☐	H H : M M	Yes ☐<br>No ☐	______	H H : M M
___________	______	Yes ☐<br>No ☐	D D M M M Y Y Y Y	H H : M M	Yes ☐<br>No ☐	H H : M M	Yes ☐<br>No ☐	______	H H : M M
___________	______	Yes ☐<br>No ☐	D D M M M Y Y Y Y	H H : M M	Yes ☐<br>No ☐	H H : M M	Yes ☐<br>No ☐	______	H H : M M

CONCOMITANT MEDICATIONS – DAY 28

Medication name	Formulation	Dose	Units	Frequency	Route	Date started	Date stopped	Ongoing?	Indication
___________	______	____	____	______	______	D D M M M Y Y Y Y	D D M M M Y Y Y Y	Yes ☐ No ☐	___________
___________	______	____	____	______	______	D D M M M Y Y Y Y	D D M M M Y Y Y Y	Yes ☐ No ☐	___________
___________	______	____	____	______	______	D D M M M Y Y Y Y	D D M M M Y Y Y Y	Yes ☐ No ☐	___________
___________	______	____	____	______	______	D D M M M Y Y Y Y	D D M M M Y Y Y Y	Yes ☐ No ☐	___________
___________	______	____	____	______	______	D D M M M Y Y Y Y	D D M M M Y Y Y Y	Yes ☐ No ☐	___________
___________	______	____	____	______	______	D D M M M Y Y Y Y	D D M M M Y Y Y Y	Yes ☐ No ☐	___________
___________	______	____	____	______	______	D D M M M Y Y Y Y	D D M M M Y Y Y Y	Yes ☐ No ☐	___________
___________	______	____	____	______	______	D D M M M Y Y Y Y	D D M M M Y Y Y Y	Yes ☐ No ☐	___________
___________	______	____	____	______	______	D D M M M Y Y Y Y	D D M M M Y Y Y Y	Yes ☐ No ☐	___________
___________	______	____	____	______	______	D D M M M Y Y Y Y	D D M M M Y Y Y Y	Yes ☐ No ☐	___________
___________	______	____	____	______	______	D D M M M Y Y Y Y	D D M M M Y Y Y Y	Yes ☐ No ☐	___________

ADVERSE EVENTS – DAY 28

Event Name	Intensity			Onset Date	End Date	Ongoing?	Action Taken with Study Drug	Relationship to Study drug
							1 Recovered<br>2 Recovering<br>3 Recovering with sequelae<br>4 Continuing<br>5 Fatal<br>99 Not Known	1 Certain<br>4 Unlikely<br>2 Probable<br>5 Not related<br>3 Possible<br>6 Unclassified
________	☐ 1 Mild	☐ 2 Moderate	☐ 3 Severe	D D M M M Y Y Y Y	D D M M M Y Y Y Y	Yes ☐<br>No ☐	________	________
________	☐ 1 Mild	☐ 2 Moderate	☐ 3 Severe	D D M M M Y Y Y Y	D D M M M Y Y Y Y	Yes ☐<br>No ☐	________	________
________	☐ 1 Mild	☐ 2 Moderate	☐ 3 Severe	D D M M M Y Y Y Y	D D M M M Y Y Y Y	Yes ☐<br>No ☐	________	________
________	☐ 1 Mild	☐ 2 Moderate	☐ 3 Severe	D D M M M Y Y Y Y	D D M M M Y Y Y Y	Yes ☐<br>No ☐	________	________
________	☐ 1 Mild	☐ 2 Moderate	☐ 3 Severe	D D M M M Y Y Y Y	D D M M M Y Y Y Y	Yes ☐<br>No ☐	________	________
________	☐ 1 Mild	☐ 2 Moderate	☐ 3 Severe	D D M M M Y Y Y Y	D D M M M Y Y Y Y	Yes ☐<br>No ☐	________	________
________	☐ 1 Mild	☐ 2 Moderate	☐ 3 Severe	D D M M M Y Y Y Y	D D M M M Y Y Y Y	Yes ☐<br>No ☐	________	________
________	☐ 1 Mild	☐ 2 Moderate	☐ 3 Severe	D D M M M Y Y Y Y	D D M M M Y Y Y Y	Yes ☐<br>No ☐	________	________
________	☐ 1 Mild	☐ 2 Moderate	☐ 3 Severe	D D M M M Y Y Y Y	D D M M M Y Y Y Y	Yes ☐<br>No ☐	________	________
________	☐ 1 Mild	☐ 2 Moderate	☐ 3 Severe	D D M M M Y Y Y Y	D D M M M Y Y Y Y	Yes ☐<br>No ☐	________	________
________	☐ 1 Mild	☐ 2 Moderate	☐ 3 Severe	D D M M M Y Y Y Y	D D M M M Y Y Y Y	Yes ☐<br>No ☐	________	________

CASE REPORT FORM

CYCLE-1

Participant Number	

PHYSICAL EXAMINATION – CYCLE 1

Date	D D M M M Y Y Y Y	Time	H H : M M
Weight	. kg	Height	. cm
Temperature	. °C	Method of Recording: Axillary ☐1 Tympanic ☐2 Rectal ☐3 Oral ☐4	Heart rate bpm
Respiratory rate	bpm	Blood pressure	/ m
Hepatomegaly	☐1. Yes ☐2. No	If yes, size:	cm
Splenomegaly	☐1. Yes ☐2. No	If yes, size:	cm

HAEMATOLOGY – CYCLE 1

Date<br>D D M M M Y Y Y Y	Time 24hr<br>H H : M M	Hb (g/dL)<br>.	Hct (%)<br>.	WBC (10^9/L)<br>.
Neutrophils (%)<br>.	Lymphocytes (%)<br>.	Monocytes (%)<br>.	Eosinophils (%)<br>.	Platelets (10^9/L)<br>.

SYMPTOM CHECK – CYCLE 1

Date<br>D D M M M Y Y Y Y	Fever<br>Yes ☐<br>No ☐	Dizziness<br>Yes ☐<br>No ☐	Headache<br>Yes ☐<br>No ☐	Nausea<br>Yes ☐<br>No ☐	Anorexia<br>Yes ☐<br>No ☐	Vomiting<br>Yes ☐<br>No ☐
Time 24hr<br>H H : M M	Diarrhoea<br>Yes ☐<br>No ☐	Abdominal pain<br>Yes ☐<br>No ☐	Itching<br>Yes ☐<br>No ☐	Skin rash<br>Yes ☐<br>No ☐	Urticaria<br>Yes ☐<br>No ☐	Joint pain<br>Yes ☐<br>No ☐
Muscle pain<br>Yes ☐<br>No ☐	Palpitations<br>Yes ☐<br>No ☐	Dyspnoea<br>Yes ☐<br>No ☐	Hearing problem<br>Yes ☐<br>No ☐	Confusion<br>Yes ☐<br>No ☐	Visual blurring<br>Yes ☐<br>No ☐	Fatigue<br>Yes ☐<br>No ☐

STUDY DRUG ADMINSTRATION – CYCLE 1

Study drug	Dose	Treatment observed?	Date of dose	Time of dose	Vomited?	Time of vomit	Retreatment?	Retreatment dose	Time of retreatment
____________	_______	Yes ☐<br>No ☐	D D M M M Y Y Y Y	H H : M M	Yes ☐<br>No ☐	H H : M M	Yes ☐<br>No ☐	_______	H H : M M
____________	_______	Yes ☐<br>No ☐	D D M M M Y Y Y Y	H H : M M	Yes ☐<br>No ☐	H H : M M	Yes ☐<br>No ☐	_______	H H : M M
____________	_______	Yes ☐<br>No ☐	D D M M M Y Y Y Y	H H : M M	Yes ☐<br>No ☐	H H : M M	Yes ☐<br>No ☐	_______	H H : M M
____________	_______	Yes ☐<br>No ☐	D D M M M Y Y Y Y	H H : M M	Yes ☐<br>No ☐	H H : M M	Yes ☐<br>No ☐	_______	H H : M M
____________	_______	Yes ☐<br>No ☐	D D M M M Y Y Y Y	H H : M M	Yes ☐<br>No ☐	H H : M M	Yes ☐<br>No ☐	_______	H H : M M
____________	_______	Yes ☐<br>No ☐	D D M M M Y Y Y Y	H H : M M	Yes ☐<br>No ☐	H H : M M	Yes ☐<br>No ☐	_______	H H : M M
____________	_______	Yes ☐<br>No ☐	D D M M M Y Y Y Y	H H : M M	Yes ☐<br>No ☐	H H : M M	Yes ☐<br>No ☐	_______	H H : M M
____________	_______	Yes ☐<br>No ☐	D D M M M Y Y Y Y	H H : M M	Yes ☐<br>No ☐	H H : M M	Yes ☐<br>No ☐	_______	H H : M M
____________	_______	Yes ☐<br>No ☐	D D M M M Y Y Y Y	H H : M M	Yes ☐<br>No ☐	H H : M M	Yes ☐<br>No ☐	_______	H H : M M
____________	_______	Yes ☐<br>No ☐	D D M M M Y Y Y Y	H H : M M	Yes ☐<br>No ☐	H H : M M	Yes ☐<br>No ☐	_______	H H : M M
____________	_______	Yes ☐<br>No ☐	D D M M M Y Y Y Y	H H : M M	Yes ☐<br>No ☐	H H : M M	Yes ☐<br>No ☐	_______	H H : M M

CONCOMITANT MEDICATIONS – CYCLE 1

Medication name	Formulation	Dose	Units	Frequency	Route	Date started	Date stopped	Ongoing?	Indication
______	______	______	______	______	______	D D M M M Y Y Y Y	D D M M M Y Y Y Y	Yes ☐ No ☐	______
______	______	______	______	______	______	D D M M M Y Y Y Y	D D M M M Y Y Y Y	Yes ☐ No ☐	______
______	______	______	______	______	______	D D M M M Y Y Y Y	D D M M M Y Y Y Y	Yes ☐ No ☐	______
______	______	______	______	______	______	D D M M M Y Y Y Y	D D M M M Y Y Y Y	Yes ☐ No ☐	______
______	______	______	______	______	______	D D M M M Y Y Y Y	D D M M M Y Y Y Y	Yes ☐ No ☐	______
______	______	______	______	______	______	D D M M M Y Y Y Y	D D M M M Y Y Y Y	Yes ☐ No ☐	______
______	______	______	______	______	______	D D M M M Y Y Y Y	D D M M M Y Y Y Y	Yes ☐ No ☐	______
______	______	______	______	______	______	D D M M M Y Y Y Y	D D M M M Y Y Y Y	Yes ☐ No ☐	______
______	______	______	______	______	______	D D M M M Y Y Y Y	D D M M M Y Y Y Y	Yes ☐ No ☐	______
______	______	______	______	______	______	D D M M M Y Y Y Y	D D M M M Y Y Y Y	Yes ☐ No ☐	______
______	______	______	______	______	______	D D M M M Y Y Y Y	D D M M M Y Y Y Y	Yes ☐ No ☐	______

ADVERSE EVENTS – CYCLE 1

Event Name	Intensity			Onset Date	End Date	Ongoing?	Action Taken with Study Drug	Relationship to Study drug
							1 Recovered<br>2 Recovering<br>3 Recovering with sequelae<br>4 Continuing<br>5 Fatal<br>99 Not Known	1 Certain<br>4 Unlikely<br>2 Probable<br>5 Not related<br>3 Possible<br>6 Unclassified
______	☐1 Mild	☐2 Moderate	☐3 Severe	D D M M M Y Y Y Y	D D M M M Y Y Y Y	Yes ☐<br>No ☐	______	______
______	☐1 Mild	☐2 Moderate	☐3 Severe	D D M M M Y Y Y Y	D D M M M Y Y Y Y	Yes ☐<br>No ☐	______	______
______	☐1 Mild	☐2 Moderate	☐3 Severe	D D M M M Y Y Y Y	D D M M M Y Y Y Y	Yes ☐<br>No ☐	______	______
______	☐1 Mild	☐2 Moderate	☐3 Severe	D D M M M Y Y Y Y	D D M M M Y Y Y Y	Yes ☐<br>No ☐	______	______
______	☐1 Mild	☐2 Moderate	☐3 Severe	D D M M M Y Y Y Y	D D M M M Y Y Y Y	Yes ☐<br>No ☐	______	______
______	☐1 Mild	☐2 Moderate	☐3 Severe	D D M M M Y Y Y Y	D D M M M Y Y Y Y	Yes ☐<br>No ☐	______	______
______	☐1 Mild	☐2 Moderate	☐3 Severe	D D M M M Y Y Y Y	D D M M M Y Y Y Y	Yes ☐<br>No ☐	______	______
______	☐1 Mild	☐2 Moderate	☐3 Severe	D D M M M Y Y Y Y	D D M M M Y Y Y Y	Yes ☐<br>No ☐	______	______
______	☐1 Mild	☐2 Moderate	☐3 Severe	D D M M M Y Y Y Y	D D M M M Y Y Y Y	Yes ☐<br>No ☐	______	______
______	☐1 Mild	☐2 Moderate	☐3 Severe	D D M M M Y Y Y Y	D D M M M Y Y Y Y	Yes ☐<br>No ☐	______	______

CASE REPORT FORM

CYCLE-2

Participant Number	☐☐☐☐

PHYSICAL EXAMINATION – CYCLE 2

Date	D D M M M Y Y Y Y	Time	H H : M M
Weight	☐☐☐.☐ kg	Height	☐☐☐.☐☐ cm
Temperature	☐☐.☐ °C	Method of Recording: Axillary ☐1 Tympanic ☐2 Rectal ☐3 Oral ☐4	Heart rate ☐☐☐ bpm
Respiratory rate	☐☐ bpm	Blood pressure	☐☐☐ / ☐☐☐ m
Hepatomegaly	☐1. Yes ☐2. No	If yes, size:	☐☐ cm
Splenomegaly	☐1. Yes ☐2. No	If yes, size:	☐☐ cm

HAEMATOLOGY – CYCLE 2

Date<br>D D M M M Y Y Y Y	Time 24h<br>H H : M M	Hb (g/dL)<br>☐☐.☐	Hct (%)<br>☐☐.☐	WBC (10^9/L)<br>☐☐☐.☐
Neutrophils (%)<br>☐☐.☐	Lymphocytes (%)<br>☐☐.☐	Monocytes (%)<br>☐☐.☐	Eosinophils (%)<br>☐☐.☐	Platelets (10^9/L)<br>☐☐☐.☐

SYMPTOM CHECK – CYCLE 2

Date<br>D D M M M Y Y Y Y	Fever<br>Yes ☐<br>No ☐	Dizziness<br>Yes ☐<br>No ☐	Headache<br>Yes ☐<br>No ☐	Nausea<br>Yes ☐<br>No ☐	Anorexia<br>Yes ☐<br>No ☐	Vomiting<br>Yes ☐<br>No ☐
Time 24hr<br>H H : M M	Diarrhoea<br>Yes ☐<br>No ☐	Abdominal pain<br>Yes ☐<br>No ☐	Itching<br>Yes ☐<br>No ☐	Skin rash<br>Yes ☐<br>No ☐	Urticaria<br>Yes ☐<br>No ☐	Joint pain<br>Yes ☐<br>No ☐
Muscle pain<br>Yes ☐<br>No ☐	Palpitations<br>Yes ☐<br>No ☐	Dyspnoea<br>Yes ☐<br>No ☐	Hearing problem<br>Yes ☐<br>No ☐	Confusion<br>Yes ☐<br>No ☐	Visual blurring<br>Yes ☐<br>No ☐	Fatigue<br>Yes ☐<br>No ☐

STUDY DRUG ADMINSTRATION – CYCLE 2

Study drug	Dose	Treatment observed?	Date of dose	Time of dose	Vomited?	Time of vomit	Retreatment?	Retreatment dose	Time of retreatment
________	______	Yes ☐<br>No ☐	D D M M M Y Y Y Y	H H : M M	Yes ☐<br>No ☐	H H : M M	Yes ☐<br>No ☐	______	H H : M M
________	______	Yes ☐<br>No ☐	D D M M M Y Y Y Y	H H : M M	Yes ☐<br>No ☐	H H : M M	Yes ☐<br>No ☐	______	H H : M M
________	______	Yes ☐<br>No ☐	D D M M M Y Y Y Y	H H : M M	Yes ☐<br>No ☐	H H : M M	Yes ☐<br>No ☐	______	H H : M M
________	______	Yes ☐<br>No ☐	D D M M M Y Y Y Y	H H : M M	Yes ☐<br>No ☐	H H : M M	Yes ☐<br>No ☐	______	H H : M M
________	______	Yes ☐<br>No ☐	D D M M M Y Y Y Y	H H : M M	Yes ☐<br>No ☐	H H : M M	Yes ☐<br>No ☐	______	H H : M M
________	______	Yes ☐<br>No ☐	D D M M M Y Y Y Y	H H : M M	Yes ☐<br>No ☐	H H : M M	Yes ☐<br>No ☐	______	H H : M M
________	______	Yes ☐<br>No ☐	D D M M M Y Y Y Y	H H : M M	Yes ☐<br>No ☐	H H : M M	Yes ☐<br>No ☐	______	H H : M M
________	______	Yes ☐<br>No ☐	D D M M M Y Y Y Y	H H : M M	Yes ☐<br>No ☐	H H : M M	Yes ☐<br>No ☐	______	H H : M M
________	______	Yes ☐<br>No ☐	D D M M M Y Y Y Y	H H : M M	Yes ☐<br>No ☐	H H : M M	Yes ☐<br>No ☐	______	H H : M M
________	______	Yes ☐<br>No ☐	D D M M M Y Y Y Y	H H : M M	Yes ☐<br>No ☐	H H : M M	Yes ☐<br>No ☐	______	H H : M M
________	______	Yes ☐<br>No ☐	D D M M M Y Y Y Y	H H : M M	Yes ☐<br>No ☐	H H : M M	Yes ☐<br>No ☐	______	H H : M M

CONCOMITANT MEDICATIONS – CYCLE 2

Medication name	Formulation	Dose	Units	Frequency	Route	Date started	Date stopped	Ongoing?	Indication
________	______	____	____	______	______	D D M M M Y Y Y Y	D D M M M Y Y Y Y	Yes ☐ No ☐	________
________	______	____	____	______	______	D D M M M Y Y Y Y	D D M M M Y Y Y Y	Yes ☐ No ☐	________
________	______	____	____	______	______	D D M M M Y Y Y Y	D D M M M Y Y Y Y	Yes ☐ No ☐	________
________	______	____	____	______	______	D D M M M Y Y Y Y	D D M M M Y Y Y Y	Yes ☐ No ☐	________
________	______	____	____	______	______	D D M M M Y Y Y Y	D D M M M Y Y Y Y	Yes ☐ No ☐	________
________	______	____	____	______	______	D D M M M Y Y Y Y	D D M M M Y Y Y Y	Yes ☐ No ☐	________
________	______	____	____	______	______	D D M M M Y Y Y Y	D D M M M Y Y Y Y	Yes ☐ No ☐	________
________	______	____	____	______	______	D D M M M Y Y Y Y	D D M M M Y Y Y Y	Yes ☐ No ☐	________
________	______	____	____	______	______	D D M M M Y Y Y Y	D D M M M Y Y Y Y	Yes ☐ No ☐	________
________	______	____	____	______	______	D D M M M Y Y Y Y	D D M M M Y Y Y Y	Yes ☐ No ☐	________
________	______	____	____	______	______	D D M M M Y Y Y Y	D D M M M Y Y Y Y	Yes ☐ No ☐	________

ADVERSE EVENTS – CYCLE 2

Event Name	Intensity			Onset Date	End Date	Ongoing?	Action Taken with Study Drug	Relationship to Study drug
							1 Recovered<br>2 Recovering<br>3 Recovering with sequelae<br>4 Continuing<br>5 Fatal<br>99 Not Known	1 Certain<br>4 Unlikely<br>2 Probable<br>5 Not related<br>3 Possible<br>6 Unclassified
____________	☐1 Mild	☐2 Moderate	☐3 Severe	D D M M M Y Y Y Y	D D M M M Y Y Y Y	Yes ☐<br>No ☐	____________	____________
____________	☐1 Mild	☐2 Moderate	☐3 Severe	D D M M M Y Y Y Y	D D M M M Y Y Y Y	Yes ☐<br>No ☐	____________	____________
____________	☐1 Mild	☐2 Moderate	☐3 Severe	D D M M M Y Y Y Y	D D M M M Y Y Y Y	Yes ☐<br>No ☐	____________	____________
____________	☐1 Mild	☐2 Moderate	☐3 Severe	D D M M M Y Y Y Y	D D M M M Y Y Y Y	Yes ☐<br>No ☐	____________	____________
____________	☐1 Mild	☐2 Moderate	☐3 Severe	D D M M M Y Y Y Y	D D M M M Y Y Y Y	Yes ☐<br>No ☐	____________	____________
____________	☐1 Mild	☐2 Moderate	☐3 Severe	D D M M M Y Y Y Y	D D M M M Y Y Y Y	Yes ☐<br>No ☐	____________	____________
____________	☐1 Mild	☐2 Moderate	☐3 Severe	D D M M M Y Y Y Y	D D M M M Y Y Y Y	Yes ☐<br>No ☐	____________	____________
____________	☐1 Mild	☐2 Moderate	☐3 Severe	D D M M M Y Y Y Y	D D M M M Y Y Y Y	Yes ☐<br>No ☐	____________	____________
____________	☐1 Mild	☐2 Moderate	☐3 Severe	D D M M M Y Y Y Y	D D M M M Y Y Y Y	Yes ☐<br>No ☐	____________	____________
____________	☐1 Mild	☐2 Moderate	☐3 Severe	D D M M M Y Y Y Y	D D M M M Y Y Y Y	Yes ☐<br>No ☐	____________	____________

CASE REPORT FORM

CYCLE-3

Participant Number	

PHYSICAL EXAMINATION – CYCLE 3

Date	D D M M M Y Y Y Y	Time	H H : M M
Weight	. kg	Height	. cm
Temperature	. °C	Method of Recording: Axillary ☐1 Tympanic ☐2 Rectal ☐3 Oral ☐4	Heart rate bpm
Respiratory rate	bpm	Blood pressure	/ n
Hepatomegaly	☐1. Yes ☐2. No	If yes, size:	cm
Splenomegaly	☐1. Yes ☐2. No	If yes, size:	cm

HAEMATOLOGY – CYCLE 3

Date	Time 24hr	Hb (g/dL)	Hct (%)	WBC (10^9/L)
D D M M M Y Y Y Y	H H : M M	.	.	.
Neutrophils (%)	Lymphocytes (%)	Monocytes (%)	Eosinophils (%)	Platelets (10^9/L)
.	.	.	.	.

SYMPTOM CHECK – CYCLE 3

Date	Fever	Dizziness	Headache	Nausea	Anorexia	Vomiting
D D M M M Y Y Y Y	Yes ☐ No ☐	Yes ☐ No ☐	Yes ☐ No ☐	Yes ☐ No ☐	Yes ☐ No ☐	Yes ☐ No ☐

Time 24hr	Diarrhoea	Abdominal pain	Itching	Skin rash	Urticaria	Joint pain
H H : M M	Yes ☐ No ☐	Yes ☐ No ☐	Yes ☐ No ☐	Yes ☐ No ☐	Yes ☐ No ☐	Yes ☐ No ☐

Muscle pain	Palpitations	Dyspnoea	Hearing problem	Confusion	Visual blurring	Fatigue
Yes ☐ No ☐	Yes ☐ No ☐	Yes ☐ No ☐	Yes ☐ No ☐	Yes ☐ No ☐	Yes ☐ No ☐	Yes ☐ No ☐

STUDY DRUG ADMINSTRATION – CYCLE 3

Study drug	Dose	Treatment observed?	Date of dose	Time of dose	Vomited?	Time of vomit	Retreatment?	Retreatment dose	Time of retreatment
______	______	Yes ☐ No ☐	DD MMM YYYY	HH:MM	Yes ☐ No ☐	HH:MM	Yes ☐ No ☐	______	HH:MM
______	______	Yes ☐ No ☐	DD MMM YYYY	HH:MM	Yes ☐ No ☐	HH:MM	Yes ☐ No ☐	______	HH:MM
______	______	Yes ☐ No ☐	DD MMM YYYY	HH:MM	Yes ☐ No ☐	HH:MM	Yes ☐ No ☐	______	HH:MM
______	______	Yes ☐ No ☐	DD MMM YYYY	HH:MM	Yes ☐ No ☐	HH:MM	Yes ☐ No ☐	______	HH:MM
______	______	Yes ☐ No ☐	DD MMM YYYY	HH:MM	Yes ☐ No ☐	HH:MM	Yes ☐ No ☐	______	HH:MM
______	______	Yes ☐ No ☐	DD MMM YYYY	HH:MM	Yes ☐ No ☐	HH:MM	Yes ☐ No ☐	______	HH:MM
______	______	Yes ☐ No ☐	DD MMM YYYY	HH:MM	Yes ☐ No ☐	HH:MM	Yes ☐ No ☐	______	HH:MM
______	______	Yes ☐ No ☐	DD MMM YYYY	HH:MM	Yes ☐ No ☐	HH:MM	Yes ☐ No ☐	______	HH:MM
______	______	Yes ☐ No ☐	DD MMM YYYY	HH:MM	Yes ☐ No ☐	HH:MM	Yes ☐ No ☐	______	HH:MM
______	______	Yes ☐ No ☐	DD MMM YYYY	HH:MM	Yes ☐ No ☐	HH:MM	Yes ☐ No ☐	______	HH:MM
______	______	Yes ☐ No ☐	DD MMM YYYY	HH:MM	Yes ☐ No ☐	HH:MM	Yes ☐ No ☐	______	HH:MM

CONCOMITANT MEDICATIONS – CYCLE 3

Medication name	Formulation	Dose	Units	Frequency	Route	Date started	Date stopped	Ongoing?	Indication
______	______	______	______	______	______	D D M M M Y Y Y Y	D D M M M Y Y Y Y	Yes ☐ No ☐	______
______	______	______	______	______	______	D D M M M Y Y Y Y	D D M M M Y Y Y Y	Yes ☐ No ☐	______
______	______	______	______	______	______	D D M M M Y Y Y Y	D D M M M Y Y Y Y	Yes ☐ No ☐	______
______	______	______	______	______	______	D D M M M Y Y Y Y	D D M M M Y Y Y Y	Yes ☐ No ☐	______
______	______	______	______	______	______	D D M M M Y Y Y Y	D D M M M Y Y Y Y	Yes ☐ No ☐	______
______	______	______	______	______	______	D D M M M Y Y Y Y	D D M M M Y Y Y Y	Yes ☐ No ☐	______
______	______	______	______	______	______	D D M M M Y Y Y Y	D D M M M Y Y Y Y	Yes ☐ No ☐	______
______	______	______	______	______	______	D D M M M Y Y Y Y	D D M M M Y Y Y Y	Yes ☐ No ☐	______
______	______	______	______	______	______	D D M M M Y Y Y Y	D D M M M Y Y Y Y	Yes ☐ No ☐	______
______	______	______	______	______	______	D D M M M Y Y Y Y	D D M M M Y Y Y Y	Yes ☐ No ☐	______
______	______	______	______	______	______	D D M M M Y Y Y Y	D D M M M Y Y Y Y	Yes ☐ No ☐	______

ADVERSE EVENTS – CYCLE 3

Event Name	Intensity			Onset Date	End Date	Ongoing?	Action Taken with Study Drug<br>1Recovered<br>2Recovering<br>3Recovering with sequelae<br>4Continuing<br>5Fatal<br>99Not Known	Relationship to Study drug<br>1Certain<br>4Unlikely<br>2Probable<br>5Not related<br>3Possible<br>6Unclassified
____________	☐1 Mild	☐2 Moderate	☐3 Severe	D D M M M Y Y Y Y	D D M M M Y Y Y Y	Yes ☐ No ☐	____________	____________
____________	☐1 Mild	☐2 Moderate	☐3 Severe	D D M M M Y Y Y Y	D D M M M Y Y Y Y	Yes ☐ No ☐	____________	____________
____________	☐1 Mild	☐2 Moderate	☐3 Severe	D D M M M Y Y Y Y	D D M M M Y Y Y Y	Yes ☐ No ☐	____________	____________
____________	☐1 Mild	☐2 Moderate	☐3 Severe	D D M M M Y Y Y Y	D D M M M Y Y Y Y	Yes ☐ No ☐	____________	____________
____________	☐1 Mild	☐2 Moderate	☐3 Severe	D D M M M Y Y Y Y	D D M M M Y Y Y Y	Yes ☐ No ☐	____________	____________
____________	☐1 Mild	☐2 Moderate	☐3 Severe	D D M M M Y Y Y Y	D D M M M Y Y Y Y	Yes ☐ No ☐	____________	____________
____________	☐1 Mild	☐2 Moderate	☐3 Severe	D D M M M Y Y Y Y	D D M M M Y Y Y Y	Yes ☐ No ☐	____________	____________
____________	☐1 Mild	☐2 Moderate	☐3 Severe	D D M M M Y Y Y Y	D D M M M Y Y Y Y	Yes ☐ No ☐	____________	____________
____________	☐1 Mild	☐2 Moderate	☐3 Severe	D D M M M Y Y Y Y	D D M M M Y Y Y Y	Yes ☐ No ☐	____________	____________
____________	☐1 Mild	☐2 Moderate	☐3 Severe	D D M M M Y Y Y Y	D D M M M Y Y Y Y	Yes ☐ No ☐	____________	____________

CASE REPORT FORM

CYCLE-4

Participant Number	

PHYSICAL EXAMINATION – CYCLE 4

Date	DD MMM YYYY	Time	HH : MM
Weight	. kg	Height	. cm
Temperature	. °C — Method of Recording: Axillary ☐1 Tympanic ☐2 Rectal ☐3 Oral ☐4	Heart rate	bpm
Respiratory rate	bpm	Blood pressure	/ m
Hepatomegaly	☐1. Yes ☐2. No	If yes, size:	cm
Splenomegaly	☐1. Yes ☐2. No	If yes, size:	cm

HAEMATOLOGY – CYCLE 4

Date<br>DD MMM YYYY	Time 24hr<br>HH : MM	Hb (g/dL)<br>.	Hct (%)<br>.	WBC (10^9/L)<br>.
Neutrophils (%)<br>.	Lymphocytes (%)<br>.	Monocytes (%)<br>.	Eosinophils (%)<br>.	Platelets (10^9/L)<br>.

SYMPTOM CHECK – CYCLE 4

Date<br>DD MMM YYYY	Fever<br>Yes ☐<br>No ☐	Dizziness<br>Yes ☐<br>No ☐	Headache<br>Yes ☐<br>No ☐	Nausea<br>Yes ☐<br>No ☐	Anorexia<br>Yes ☐<br>No ☐	Vomiting<br>Yes ☐<br>No ☐
Time 24hr<br>HH : MM	Diarrhoea<br>Yes ☐<br>No ☐	Abdominal pain<br>Yes ☐<br>No ☐	Itching<br>Yes ☐<br>No ☐	Skin rash<br>Yes ☐<br>No ☐	Urticaria<br>Yes ☐<br>No ☐	Joint pain<br>Yes ☐<br>No ☐
Muscle pain<br>Yes ☐<br>No ☐	Palpitations<br>Yes ☐<br>No ☐	Dyspnoea<br>Yes ☐<br>No ☐	Hearing problem<br>Yes ☐<br>No ☐	Confusion<br>Yes ☐<br>No ☐	Visual blurring<br>Yes ☐<br>No ☐	Fatigue<br>Yes ☐<br>No ☐

STUDY DRUG ADMINSTRATION – CYCLE 4

Study drug	Dose	Treatment observed?	Date of dose	Time of dose	Vomited?	Time of vomit	Retreatment?	Retreatment dose	Time of retreatment
___________	______	Yes ☐ No ☐	D D M M M Y Y Y Y	H H : M M	Yes ☐ No ☐	H H : M M	Yes ☐ No ☐	______	H H : M M
___________	______	Yes ☐ No ☐	D D M M M Y Y Y Y	H H : M M	Yes ☐ No ☐	H H : M M	Yes ☐ No ☐	______	H H : M M
___________	______	Yes ☐ No ☐	D D M M M Y Y Y Y	H H : M M	Yes ☐ No ☐	H H : M M	Yes ☐ No ☐	______	H H : M M
___________	______	Yes ☐ No ☐	D D M M M Y Y Y Y	H H : M M	Yes ☐ No ☐	H H : M M	Yes ☐ No ☐	______	H H : M M
___________	______	Yes ☐ No ☐	D D M M M Y Y Y Y	H H : M M	Yes ☐ No ☐	H H : M M	Yes ☐ No ☐	______	H H : M M
___________	______	Yes ☐ No ☐	D D M M M Y Y Y Y	H H : M M	Yes ☐ No ☐	H H : M M	Yes ☐ No ☐	______	H H : M M
___________	______	Yes ☐ No ☐	D D M M M Y Y Y Y	H H : M M	Yes ☐ No ☐	H H : M M	Yes ☐ No ☐	______	H H : M M
___________	______	Yes ☐ No ☐	D D M M M Y Y Y Y	H H : M M	Yes ☐ No ☐	H H : M M	Yes ☐ No ☐	______	H H : M M
___________	______	Yes ☐ No ☐	D D M M M Y Y Y Y	H H : M M	Yes ☐ No ☐	H H : M M	Yes ☐ No ☐	______	H H : M M
___________	______	Yes ☐ No ☐	D D M M M Y Y Y Y	H H : M M	Yes ☐ No ☐	H H : M M	Yes ☐ No ☐	______	H H : M M
___________	______	Yes ☐ No ☐	D D M M M Y Y Y Y	H H : M M	Yes ☐ No ☐	H H : M M	Yes ☐ No ☐	______	H H : M M

CONCOMITANT MEDICATIONS – CYCLE 4

Medication name	Formulation	Dose	Units	Frequency	Route	Date started	Date stopped	Ongoing?	Indication
						D D M M M Y Y Y Y	D D M M M Y Y Y Y	Yes ☐ No ☐	
						D D M M M Y Y Y Y	D D M M M Y Y Y Y	Yes ☐ No ☐	
						D D M M M Y Y Y Y	D D M M M Y Y Y Y	Yes ☐ No ☐	
						D D M M M Y Y Y Y	D D M M M Y Y Y Y	Yes ☐ No ☐	
						D D M M M Y Y Y Y	D D M M M Y Y Y Y	Yes ☐ No ☐	
						D D M M M Y Y Y Y	D D M M M Y Y Y Y	Yes ☐ No ☐	
						D D M M M Y Y Y Y	D D M M M Y Y Y Y	Yes ☐ No ☐	
						D D M M M Y Y Y Y	D D M M M Y Y Y Y	Yes ☐ No ☐	
						D D M M M Y Y Y Y	D D M M M Y Y Y Y	Yes ☐ No ☐	
						D D M M M Y Y Y Y	D D M M M Y Y Y Y	Yes ☐ No ☐	
						D D M M M Y Y Y Y	D D M M M Y Y Y Y	Yes ☐ No ☐	

ADVERSE EVENTS – CYCLE 4

Event Name	Intensity			Onset Date	End Date	Ongoing?	Action Taken with Study Drug<br>1 Recovered<br>2 Recovering<br>3 Recovering with sequelae<br>4 Continuing<br>5 Fatal<br>99 Not Known	Relationship to Study drug<br>1 Certain<br>4 Unlikely<br>2 Probable<br>5 Not related<br>3 Possible<br>6 Unclassified
___________	☐1 Mild	☐2 Moderate	☐3 Severe	D D M M M Y Y Y Y	D D M M M Y Y Y Y	Yes ☐<br>No ☐	___________	___________
___________	☐1 Mild	☐2 Moderate	☐3 Severe	D D M M M Y Y Y Y	D D M M M Y Y Y Y	Yes ☐<br>No ☐	___________	___________
___________	☐1 Mild	☐2 Moderate	☐3 Severe	D D M M M Y Y Y Y	D D M M M Y Y Y Y	Yes ☐<br>No ☐	___________	___________
___________	☐1 Mild	☐2 Moderate	☐3 Severe	D D M M M Y Y Y Y	D D M M M Y Y Y Y	Yes ☐<br>No ☐	___________	___________
___________	☐1 Mild	☐2 Moderate	☐3 Severe	D D M M M Y Y Y Y	D D M M M Y Y Y Y	Yes ☐<br>No ☐	___________	___________
___________	☐1 Mild	☐2 Moderate	☐3 Severe	D D M M M Y Y Y Y	D D M M M Y Y Y Y	Yes ☐<br>No ☐	___________	___________
___________	☐1 Mild	☐2 Moderate	☐3 Severe	D D M M M Y Y Y Y	D D M M M Y Y Y Y	Yes ☐<br>No ☐	___________	___________
___________	☐1 Mild	☐2 Moderate	☐3 Severe	D D M M M Y Y Y Y	D D M M M Y Y Y Y	Yes ☐<br>No ☐	___________	___________
___________	☐1 Mild	☐2 Moderate	☐3 Severe	D D M M M Y Y Y Y	D D M M M Y Y Y Y	Yes ☐<br>No ☐	___________	___________
___________	☐1 Mild	☐2 Moderate	☐3 Severe	D D M M M Y Y Y Y	D D M M M Y Y Y Y	Yes ☐<br>No ☐	___________	___________

CASE REPORT FORM

FIRST FOLLOW-UP

Participant Number	

PHYSICAL EXAMINATION – WEEK 12

Date	D D M M M Y Y Y Y		Time	H H : M M	
Weight	. kg		Height	. cm	
Temperature	. °C	Method of Recording: Axillary ☐1 Tympanic ☐2 Rectal ☐3 Oral ☐4		Heart rate	bpm
Respiratory rate	bpm		Blood pressure	/ m	
Hepatomegaly	☐1. Yes ☐2. No		If yes, size:	cm	
Splenomegaly	☐1. Yes ☐2. No		If yes, size:	cm	

SYMPTOM CHECK – WEEK 12

Date D D M M M Y Y Y Y	Fever Yes ☐ No ☐	Dizziness Yes ☐ No ☐	Headache Yes ☐ No ☐	Nausea Yes ☐ No ☐	Anorexia Yes ☐ No ☐	Vomiting Yes ☐ No ☐
Time 24hr H H : M M	Diarrhoea Yes ☐ No ☐	Abdominal pain Yes ☐ No ☐	Itching Yes ☐ No ☐	Skin rash Yes ☐ No ☐	Urticaria Yes ☐ No ☐	Joint pain Yes ☐ No ☐
Muscle pain Yes ☐ No ☐	Palpitations Yes ☐ No ☐	Dyspnoea Yes ☐ No ☐	Hearing problem Yes ☐ No ☐	Confusion Yes ☐ No ☐	Visual blurring Yes ☐ No ☐	Fatigue Yes ☐ No ☐

CONCOMITANT MEDICATIONS – WEEK 12

Medication name	Formulation	Dose	Units	Frequency	Route	Date started	Date stopped	Ongoing?	Indication
______	______	______	______	______	______	D D M M M Y Y Y Y	D D M M M Y Y Y Y	Yes ☐ No ☐	______
______	______	______	______	______	______	D D M M M Y Y Y Y	D D M M M Y Y Y Y	Yes ☐ No ☐	______
______	______	______	______	______	______	D D M M M Y Y Y Y	D D M M M Y Y Y Y	Yes ☐ No ☐	______
______	______	______	______	______	______	D D M M M Y Y Y Y	D D M M M Y Y Y Y	Yes ☐ No ☐	______
______	______	______	______	______	______	D D M M M Y Y Y Y	D D M M M Y Y Y Y	Yes ☐ No ☐	______
______	______	______	______	______	______	D D M M M Y Y Y Y	D D M M M Y Y Y Y	Yes ☐ No ☐	______
______	______	______	______	______	______	D D M M M Y Y Y Y	D D M M M Y Y Y Y	Yes ☐ No ☐	______
______	______	______	______	______	______	D D M M M Y Y Y Y	D D M M M Y Y Y Y	Yes ☐ No ☐	______
______	______	______	______	______	______	D D M M M Y Y Y Y	D D M M M Y Y Y Y	Yes ☐ No ☐	______
______	______	______	______	______	______	D D M M M Y Y Y Y	D D M M M Y Y Y Y	Yes ☐ No ☐	______
______	______	______	______	______	______	D D M M M Y Y Y Y	D D M M M Y Y Y Y	Yes ☐ No ☐	______

ADVERSE EVENTS – WEEK 12

Event Name	Intensity			Onset Date	End Date	Ongoing?	Action Taken with Study Drug	Relationship to Study drug
							$_1$Recovered<br>$_2$Recovering<br>$_3$Recovering with sequelae<br>$_4$Continuing<br>$_5$Fatal<br>$_{99}$Not Known	$_1$Certain<br>$_4$Unlikely<br>$_2$Probable<br>$_5$Not related<br>$_3$Possible<br>$_6$Unclassified
____________	☐$_1$ Mild	☐$_2$ Moderate	☐$_3$ Severe	D D M M M Y Y Y Y	D D M M M Y Y Y Y	Yes ☐<br>No ☐	____________	____________
____________	☐$_1$ Mild	☐$_2$ Moderate	☐$_3$ Severe	D D M M M Y Y Y Y	D D M M M Y Y Y Y	Yes ☐<br>No ☐	____________	____________
____________	☐$_1$ Mild	☐$_2$ Moderate	☐$_3$ Severe	D D M M M Y Y Y Y	D D M M M Y Y Y Y	Yes ☐<br>No ☐	____________	____________
____________	☐$_1$ Mild	☐$_2$ Moderate	☐$_3$ Severe	D D M M M Y Y Y Y	D D M M M Y Y Y Y	Yes ☐<br>No ☐	____________	____________
____________	☐$_1$ Mild	☐$_2$ Moderate	☐$_3$ Severe	D D M M M Y Y Y Y	D D M M M Y Y Y Y	Yes ☐<br>No ☐	____________	____________
____________	☐$_1$ Mild	☐$_2$ Moderate	☐$_3$ Severe	D D M M M Y Y Y Y	D D M M M Y Y Y Y	Yes ☐<br>No ☐	____________	____________
____________	☐$_1$ Mild	☐$_2$ Moderate	☐$_3$ Severe	D D M M M Y Y Y Y	D D M M M Y Y Y Y	Yes ☐<br>No ☐	____________	____________
____________	☐$_1$ Mild	☐$_2$ Moderate	☐$_3$ Severe	D D M M M Y Y Y Y	D D M M M Y Y Y Y	Yes ☐<br>No ☐	____________	____________
____________	☐$_1$ Mild	☐$_2$ Moderate	☐$_3$ Severe	D D M M M Y Y Y Y	D D M M M Y Y Y Y	Yes ☐<br>No ☐	____________	____________
____________	☐$_1$ Mild	☐$_2$ Moderate	☐$_3$ Severe	D D M M M Y Y Y Y	D D M M M Y Y Y Y	Yes ☐<br>No ☐	____________	____________

CASE REPORT FORM

SECOND FOLLOW-UP

Participant Number	

PHYSICAL EXAMINATION – WEEK 24

Date	D D M M M Y Y Y Y	Time	H H : M M
Weight	. kg	Height	. cm
Temperature	. °C	Method of Recording: Axillary ☐1 Tympanic ☐2 Rectal ☐3 Oral ☐4	Heart rate bpm
Respiratory rate	bpm	Blood pressure	/ m
Hepatomegaly	☐1. Yes ☐2. No	If yes, size:	cm
Splenomegaly	☐1. Yes ☐2. No	If yes, size:	cm

SYMPTOM CHECK – WEEK 24

Date D D M M M Y Y Y Y	Fever Yes ☐ No ☐	Dizziness Yes ☐ No ☐	Headache Yes ☐ No ☐	Nausea Yes ☐ No ☐	Anorexia Yes ☐ No ☐	Vomiting Yes ☐ No ☐
Time 24hr H H : M M	Diarrhoea Yes ☐ No ☐	Abdominal pain Yes ☐ No ☐	Itching Yes ☐ No ☐	Skin rash Yes ☐ No ☐	Urticaria Yes ☐ No ☐	Joint pain Yes ☐ No ☐
Muscle pain Yes ☐ No ☐	Palpitations Yes ☐ No ☐	Dyspnoea Yes ☐ No ☐	Hearing problem Yes ☐ No ☐	Confusion Yes ☐ No ☐	Visual blurring Yes ☐ No ☐	Fatigue Yes ☐ No ☐

CONCOMITANT MEDICATIONS – WEEK 24

Medication name	Formulation	Dose	Units	Frequency	Route	Date started	Date stopped	Ongoing?	Indication
______	______	____	____	______	______	D D M M M Y Y Y Y	D D M M M Y Y Y Y	Yes ☐ No ☐	______
______	______	____	____	______	______	D D M M M Y Y Y Y	D D M M M Y Y Y Y	Yes ☐ No ☐	______
______	______	____	____	______	______	D D M M M Y Y Y Y	D D M M M Y Y Y Y	Yes ☐ No ☐	______
______	______	____	____	______	______	D D M M M Y Y Y Y	D D M M M Y Y Y Y	Yes ☐ No ☐	______
______	______	____	____	______	______	D D M M M Y Y Y Y	D D M M M Y Y Y Y	Yes ☐ No ☐	______
______	______	____	____	______	______	D D M M M Y Y Y Y	D D M M M Y Y Y Y	Yes ☐ No ☐	______
______	______	____	____	______	______	D D M M M Y Y Y Y	D D M M M Y Y Y Y	Yes ☐ No ☐	______
______	______	____	____	______	______	D D M M M Y Y Y Y	D D M M M Y Y Y Y	Yes ☐ No ☐	______
______	______	____	____	______	______	D D M M M Y Y Y Y	D D M M M Y Y Y Y	Yes ☐ No ☐	______
______	______	____	____	______	______	D D M M M Y Y Y Y	D D M M M Y Y Y Y	Yes ☐ No ☐	______
______	______	____	____	______	______	D D M M M Y Y Y Y	D D M M M Y Y Y Y	Yes ☐ No ☐	______

ADVERSE EVENTS – WEEK 24

Event Name	Intensity			Onset Date	End Date	Ongoing?	Action Taken with Study Drug<br>1Recovered<br>2Recovering<br>3Recovering with sequelae<br>4Continuing<br>5Fatal<br>99Not Known	Relationship to Study drug<br>1Certain<br>4Unlikely<br>2Probable<br>5Not related<br>3Possible<br>6Unclassified
________	☐1 Mild	☐2 Moderate	☐3 Severe	D D M M M Y Y Y Y	D D M M M Y Y Y Y	Yes ☐ No ☐	________	________
________	☐1 Mild	☐2 Moderate	☐3 Severe	D D M M M Y Y Y Y	D D M M M Y Y Y Y	Yes ☐ No ☐	________	________
________	☐1 Mild	☐2 Moderate	☐3 Severe	D D M M M Y Y Y Y	D D M M M Y Y Y Y	Yes ☐ No ☐	________	________
________	☐1 Mild	☐2 Moderate	☐3 Severe	D D M M M Y Y Y Y	D D M M M Y Y Y Y	Yes ☐ No ☐	________	________
________	☐1 Mild	☐2 Moderate	☐3 Severe	D D M M M Y Y Y Y	D D M M M Y Y Y Y	Yes ☐ No ☐	________	________
________	☐1 Mild	☐2 Moderate	☐3 Severe	D D M M M Y Y Y Y	D D M M M Y Y Y Y	Yes ☐ No ☐	________	________
________	☐1 Mild	☐2 Moderate	☐3 Severe	D D M M M Y Y Y Y	D D M M M Y Y Y Y	Yes ☐ No ☐	________	________
________	☐1 Mild	☐2 Moderate	☐3 Severe	D D M M M Y Y Y Y	D D M M M Y Y Y Y	Yes ☐ No ☐	________	________
________	☐1 Mild	☐2 Moderate	☐3 Severe	D D M M M Y Y Y Y	D D M M M Y Y Y Y	Yes ☐ No ☐	________	________
________	☐1 Mild	☐2 Moderate	☐3 Severe	D D M M M Y Y Y Y	D D M M M Y Y Y Y	Yes ☐ No ☐	________	________

CASE REPORT FORM

THIRD FOLLOW-UP

Participant Number	

PHYSICAL EXAMINATION – WEEK 36

Date	D D M M M Y Y Y Y	Time	H H : M M
Weight	. kg	Height	. cm
Temperature	. °C<br>Method of Recording: Axillary ☐1 Tympanic ☐2 Rectal ☐3 Oral ☐4	Heart rate	bpm
Respiratory rate	bpm	Blood pressure	/ n
Hepatomegaly	☐1. Yes ☐2. No	If yes, size:	cm
Splenomegaly	☐1. Yes ☐2. No	If yes, size:	cm

SYMPTOM CHECK – WEEK 36

Date<br>D D M M M Y Y Y Y	Fever<br>Yes ☐<br>No ☐	Dizziness<br>Yes ☐<br>No ☐	Headache<br>Yes ☐<br>No ☐	Nausea<br>Yes ☐<br>No ☐	Anorexia<br>Yes ☐<br>No ☐	Vomiting<br>Yes ☐<br>No ☐
Time 24hr<br>H H : M M	Diarrhoea<br>Yes ☐<br>No ☐	Abdominal pain<br>Yes ☐<br>No ☐	Itching<br>Yes ☐<br>No ☐	Skin rash<br>Yes ☐<br>No ☐	Urticaria<br>Yes ☐<br>No ☐	Joint pain<br>Yes ☐<br>No ☐

Muscle pain<br>Yes ☐<br>No ☐	Palpitations<br>Yes ☐<br>No ☐	Dyspnoea<br>Yes ☐<br>No ☐	Hearing problem<br>Yes ☐<br>No ☐	Confusion<br>Yes ☐<br>No ☐	Visual blurring<br>Yes ☐<br>No ☐	Fatigue<br>Yes ☐<br>No ☐

CONCOMITANT MEDICATIONS – WEEK 36

Medication name	Formulation	Dose	Units	Frequency	Route	Date started	Date stopped	Ongoing?	Indication
						D D M M M Y Y Y Y	D D M M M Y Y Y Y	Yes ☐<br>No ☐	
						D D M M M Y Y Y Y	D D M M M Y Y Y Y	Yes ☐<br>No ☐	
						D D M M M Y Y Y Y	D D M M M Y Y Y Y	Yes ☐<br>No ☐	
						D D M M M Y Y Y Y	D D M M M Y Y Y Y	Yes ☐<br>No ☐	
						D D M M M Y Y Y Y	D D M M M Y Y Y Y	Yes ☐<br>No ☐	
						D D M M M Y Y Y Y	D D M M M Y Y Y Y	Yes ☐<br>No ☐	
						D D M M M Y Y Y Y	D D M M M Y Y Y Y	Yes ☐<br>No ☐	
						D D M M M Y Y Y Y	D D M M M Y Y Y Y	Yes ☐<br>No ☐	
						D D M M M Y Y Y Y	D D M M M Y Y Y Y	Yes ☐<br>No ☐	
						D D M M M Y Y Y Y	D D M M M Y Y Y Y	Yes ☐<br>No ☐	
						D D M M M Y Y Y Y	D D M M M Y Y Y Y	Yes ☐<br>No ☐	

ADVERSE EVENTS – WEEK 36

Event Name	Intensity			Onset Date	End Date	Ongoing?	Action Taken with Study Drug<br>1Recovered<br>2Recovering<br>3Recovering with sequelae<br>4Continuing<br>5Fatal<br>99Not Known	Relationship to Study drug<br>1Certain<br>4Unlikely<br>2Probable<br>5Not related<br>3Possible<br>6Unclassified
________	☐1 Mild	☐2 Moderate	☐3 Severe	D D M M M Y Y Y Y	D D M M M Y Y Y Y	Yes ☐<br>No ☐	________	________
________	☐1 Mild	☐2 Moderate	☐3 Severe	D D M M M Y Y Y Y	D D M M M Y Y Y Y	Yes ☐<br>No ☐	________	________
________	☐1 Mild	☐2 Moderate	☐3 Severe	D D M M M Y Y Y Y	D D M M M Y Y Y Y	Yes ☐<br>No ☐	________	________
________	☐1 Mild	☐2 Moderate	☐3 Severe	D D M M M Y Y Y Y	D D M M M Y Y Y Y	Yes ☐<br>No ☐	________	________
________	☐1 Mild	☐2 Moderate	☐3 Severe	D D M M M Y Y Y Y	D D M M M Y Y Y Y	Yes ☐<br>No ☐	________	________
________	☐1 Mild	☐2 Moderate	☐3 Severe	D D M M M Y Y Y Y	D D M M M Y Y Y Y	Yes ☐<br>No ☐	________	________
________	☐1 Mild	☐2 Moderate	☐3 Severe	D D M M M Y Y Y Y	D D M M M Y Y Y Y	Yes ☐<br>No ☐	________	________
________	☐1 Mild	☐2 Moderate	☐3 Severe	D D M M M Y Y Y Y	D D M M M Y Y Y Y	Yes ☐<br>No ☐	________	________
________	☐1 Mild	☐2 Moderate	☐3 Severe	D D M M M Y Y Y Y	D D M M M Y Y Y Y	Yes ☐<br>No ☐	________	________
________	☐1 Mild	☐2 Moderate	☐3 Severe	D D M M M Y Y Y Y	D D M M M Y Y Y Y	Yes ☐<br>No ☐	________	________

CASE REPORT FORM

FOURTH FOLLOW-UP

Participant Number	☐☐☐☐

PHYSICAL EXAMINATION – WEEK 48

Date	D D M M M Y Y Y Y	Time	H H : M M
Weight	___ . _ kg	Height	___ . _ cm
Temperature	__ . _ °C	Method of Recording: Axillary ☐1 Tympanic ☐2 Rectal ☐3 Oral ☐4	Heart rate ___ bpm
Respiratory rate	__ bpm	Blood pressure	___ / ___ m
Hepatomegaly	☐1. Yes ☐2. No	If yes, size:	__ cm
Splenomegaly	☐1. Yes ☐2. No	If yes, size:	__ cm

SYMPTOM CHECK – WEEK 48

Date D D M M M Y Y Y Y	Fever Yes ☐ No ☐	Dizziness Yes ☐ No ☐	Headache Yes ☐ No ☐	Nausea Yes ☐ No ☐	Anorexia Yes ☐ No ☐	Vomiting Yes ☐ No ☐
Time 24hr H H : M M	Diarrhoea Yes ☐ No ☐	Abdominal pain Yes ☐ No ☐	Itching Yes ☐ No ☐	Skin rash Yes ☐ No ☐	Urticaria Yes ☐ No ☐	Joint pain Yes ☐ No ☐
Muscle pain Yes ☐ No ☐	Palpitations Yes ☐ No ☐	Dyspnoea Yes ☐ No ☐	Hearing problem Yes ☐ No ☐	Confusion Yes ☐ No ☐	Visual blurring Yes ☐ No ☐	Fatigue Yes ☐ No ☐

CONCOMITANT MEDICATIONS – WEEK 48

Medication name	Formulation	Dose	Units	Frequency	Route	Date started	Date stopped	Ongoing?	Indication
						D D M M M Y Y Y Y	D D M M M Y Y Y Y	Yes ☐ No ☐	
						D D M M M Y Y Y Y	D D M M M Y Y Y Y	Yes ☐ No ☐	
						D D M M M Y Y Y Y	D D M M M Y Y Y Y	Yes ☐ No ☐	
						D D M M M Y Y Y Y	D D M M M Y Y Y Y	Yes ☐ No ☐	
						D D M M M Y Y Y Y	D D M M M Y Y Y Y	Yes ☐ No ☐	
						D D M M M Y Y Y Y	D D M M M Y Y Y Y	Yes ☐ No ☐	
						D D M M M Y Y Y Y	D D M M M Y Y Y Y	Yes ☐ No ☐	
						D D M M M Y Y Y Y	D D M M M Y Y Y Y	Yes ☐ No ☐	
						D D M M M Y Y Y Y	D D M M M Y Y Y Y	Yes ☐ No ☐	
						D D M M M Y Y Y Y	D D M M M Y Y Y Y	Yes ☐ No ☐	
						D D M M M Y Y Y Y	D D M M M Y Y Y Y	Yes ☐ No ☐	

ADVERSE EVENTS – WEEK 48								
**Event Name**	**Intensity**			**Onset Date**	**End Date**	**Ongoing?**	**Action Taken with Study Drug**<br>1Recovered<br>2Recovering<br>3Recovering with sequelae<br>4Continuing<br>5Fatal<br>99Not Known	**Relationship to Study drug**<br>1Certain<br>4Unlikely<br>2Probable<br>5Not related<br>3Possible<br>6Unclassified
____________	☐1 Mild	☐2 Moderate	☐3 Severe	D D M M M Y Y Y Y	D D M M M Y Y Y Y	Yes ☐<br>No ☐	____________	____________
____________	☐1 Mild	☐2 Moderate	☐3 Severe	D D M M M Y Y Y Y	D D M M M Y Y Y Y	Yes ☐<br>No ☐	____________	____________
____________	☐1 Mild	☐2 Moderate	☐3 Severe	D D M M M Y Y Y Y	D D M M M Y Y Y Y	Yes ☐<br>No ☐	____________	____________
____________	☐1 Mild	☐2 Moderate	☐3 Severe	D D M M M Y Y Y Y	D D M M M Y Y Y Y	Yes ☐<br>No ☐	____________	____________
____________	☐1 Mild	☐2 Moderate	☐3 Severe	D D M M M Y Y Y Y	D D M M M Y Y Y Y	Yes ☐<br>No ☐	____________	____________
____________	☐1 Mild	☐2 Moderate	☐3 Severe	D D M M M Y Y Y Y	D D M M M Y Y Y Y	Yes ☐<br>No ☐	____________	____________
____________	☐1 Mild	☐2 Moderate	☐3 Severe	D D M M M Y Y Y Y	D D M M M Y Y Y Y	Yes ☐<br>No ☐	____________	____________
____________	☐1 Mild	☐2 Moderate	☐3 Severe	D D M M M Y Y Y Y	D D M M M Y Y Y Y	Yes ☐<br>No ☐	____________	____________
____________	☐1 Mild	☐2 Moderate	☐3 Severe	D D M M M Y Y Y Y	D D M M M Y Y Y Y	Yes ☐<br>No ☐	____________	____________
____________	☐1 Mild	☐2 Moderate	☐3 Severe	D D M M M Y Y Y Y	D D M M M Y Y Y Y	Yes ☐<br>No ☐	____________	____________

CASE REPORT FORM

OUTCOME EVALUATION

Participant Number	☐☐☐☐

FINAL STUDY OUTCOME

**Subject has completed the study?** ☐1	**Completion date :** D D M M M Y Y Y Y
**If NOT completed specify last follow up date:**	D D M M M Y Y Y Y

Reason not completed:

(Tick only **one** box)

- ☐1 Significant non-compliance
- ☐2 Drug-related AE
- ☐3 Treatment failure
- ☐4 Consent withdrawn
- ☐5 Lost to follow-up
- ☐6 Other (specify) ____________________

Remarks: ____

____

____

Investigator's Statement: I have reviewed the data recorded in this CRF and confirm that the data are complete and accurate

Investigator (Full name): ____

Investigator Signed? ☐1

Signature Date: D D M M M Y Y Y Y

~ END ~
