# Supplementary material for: Surveillance of the Genetic Signature in Circulating Tumor DNA for Guiding Adjuvant Chemotherapy in Urothelial Carcinoma: Protocol for a Pilot Randomized Controlled Trial
Source: JMIR Res Protoc. 2025 Aug 26;14:e72597. doi: 10.2196/72597 (PMC12421199; doi:10.2196/72597)
Supplement: Multimedia Appendix 5 [file resprot_v14i1e72597_app5.pdf]

Appendix 5: Assessment schedule of the study

	Screening (day 0)	Enrollment		Treatment cycles		Discontinuation	Follow-up	
		ctDNA[a] MRD[b] test (day 28)	Group allocation (day 28)	GC[c] chemotherapy arm[d] (cycles 1 to 4)	SM[e] arm[f]		Active[g] (weeks 12, 24, 36, and 48)	Long-term[h] (every 6 months)
Time window (days)	−3	−7 to +7	+35	−3 to +3	—[i]	≤0 after final dose	−14 to +14	−28 to +28
**Recruitment**								
Informed consent	✓							
Inclusion and exclusion criteria[j]	✓							

	Medical, surgical, and cancer histories, including demographic information	✓							
	Concomitant medications	✓	✓	✓	✓	✓	✓	✓	✓
**Study intervention administration**									
	GC chemotherapy				✓				
	SM					✓			
**Safety**									
	Physical examination	✓	✓		✓	✓	✓	✓	
	Height	✓							
	Weight	✓	✓		✓	✓	✓	✓	

	Vital signs: blood pressure, heart rate, and body temperature	✓	✓		✓	✓	✓	✓	
	ECOG[k] performance status	✓			✓	✓	✓		
	Hematology, chemistry	✓			✓	✓	✓		
	12-lead ECG[l]	✓							
	HIV, HBV[m], HCV[n] serology	✓							
	TB[o] test	✓							
	Pregnancy test[p]	✓	✓		✓	✓	✓		
	Adverse event				✓	✓	✓	✓	✓
**Research-related**									

	Tumor sample collection	✓							
	Blood for ctDNA MRD testing	✓		✓				✓	
	CT TAP[q,r]	✓		✓				✓	
	Patient-reported outcomes				✓	✓	✓	✓	✓
	Quality of life	✓		✓	✓	✓	✓	✓	✓
	Mental health	✓		✓	✓	✓	✓	✓	✓
	Survival status							✓	✓

[a]ctDNA: circulating tumor DNA.

[b]MRD: molecular residual disease.

[c]GC: gemcitabine plus cisplatin.

[d]Patients will be treated with 1000 mg/m^2 intravenous gemcitabine on day 1 and day 8 plus 80 mg/m^2 intravenous cisplatin on day 1 or day 2 every 3 weeks (one cycle) for 4 cycles. A reduced dose of GC will be acceptable if the patient is not suitable for a full-dose GC. For patients with a creatinine clearance rate

of 50 to 60 mL/min, a 3-cycle regimen will also be considered upon an oncologist's evaluation.

[e]SM: standard management.

[f]As a standard of care in Hong Kong, regular follow-up will be provided, and deferred chemotherapy will be initiated until radiological progression is confirmed.

[g]Participants will be followed up until radiological confirmation of cancer recurrence, death, dropout, or study completion.

[h]Alive participants will continue to be followed up every 6 months.

[i]Not applicable.

[j]For details of participant inclusion and exclusion criteria, please refer to the Methods section of the proposal.

[k]ECOG: Eastern Cooperative Oncology Group.

[l]ECG: electrocardiograph.

[m]HBV: hepatitis B virus.

[n]HCV: hepatitis C virus.

[o]TB: tuberculosis.

[p]Female only.

[q]CT TAP: computed tomography scan of thorax, abdomen, and pelvis.

[r]Performed at weeks 12, 24, and 48.
