# Supplementary material for: Surveillance of the Genetic Signature in Circulating Tumor DNA for Guiding Adjuvant Chemotherapy in Urothelial Carcinoma: Protocol for a Pilot Randomized Controlled Trial
Source: JMIR Res Protoc. 2025 Aug 26;14:e72597. doi: 10.2196/72597 (PMC12421199; doi:10.2196/72597)
Supplement: Multimedia Appendix 6 [file resprot_v14i1e72597_app6.pdf]

REFEREE'S ASSESSMENT FORM

Reference No.: 11221566

Project Title: Surveillance of the Genetic Signature in Circulating Tumor DNA for Guiding Adjuvant Chemotherapy in Urothelial Carcinoma: A Pilot Randomized Controlled Trial

PART A: REFEREE'S DETAILED REMARKS ON THE INDIVIDUAL SECTIONS OF THE GRANT APPLICATION

1. **Originality and Impact** What is the importance of the proposed research in terms of its originality and potential impact in the area under study? How will the research findings benefit patients and/or the healthcare system? Will the research findings improve patient care, population health, influence clinical practice and/or health services management, or inform health policy in Hong Kong and elsewhere? Have the potential facilitators and barriers to this impact being achieved been identified?

Overall, the proposed research project appears to be highly relevant and important in the field of urothelial cancer management. The study aims to assess the clinical efficacy of a ctDNA-guided approach for adjuvant chemotherapy in patients with urothelial cancer. While there is already some evidence to support the use of ctDNA-guided approaches in other cancer types, the proposal suggests that there is a gap in the literature when it comes to urothelial cancer. Therefore, the proposed pilot RCT aims to fill this gap by investigating the utility of ctDNA in guiding treatment decisions for urothelial cancer patients in Hong Kong. The potential impact of the proposed research is significant, as the findings could potentially improve patient care and inform future clinical practice in Hong Kong and elsewhere. If the study demonstrates that ctDNA-guided approaches are effective in urothelial cancer patients, it could lead to more targeted and personalized treatment regimens, ultimately improving patient outcomes and quality of life. Furthermore, the findings could have broader implications for healthcare management and health policy.

2. **Research Questions, Aims and Hypotheses** How specific, clearly expressed and realistic are the research questions, aims and hypotheses?

The three aims are clearly expressed and focus on evaluating the clinical utility of ctDNA-guided approaches for adjuvant chemotherapy in urothelial cancer patients, assessing MRD surveillance through serial ctDNA testing, and providing preliminary data for a future phase III clinical trial. The two hypotheses presented are also clear and specific. The first hypothesis predicts that the use of ctDNA-guided MRD profiling will improve disease-free survival in patients receiving gemcitabine plus cisplatin chemotherapy, specifically among those who test positive for ctDNA MRD. The second hypothesis suggests that genetic profiling of ctDNA for detecting MRD will allow for real-time recurrence monitoring with a lead time prior to radiological relapse. Overall, the research questions, aims, and hypotheses are well-defined and provide a clear framework for the proposed study. They are realistic and achievable, and if the study is successful, the findings could have significant clinical implications.

3. **Subjects and Study Methodology** (i) Is the proposed design and methodology appropriate for the study? (ii) Are sample sizes clear, justified, adequate and realistic? (iii) Are any preliminary data available? (iv) How feasible is the proposed timeframe? (v) Please also provide comments on the following (where applicable):

- For proposals submitted under Advanced Medical Research (refer to Area of Project on page 1 of application), is this a clinical study which applies advanced technologies including but not limited to biotechnology in medicine, use of drugs and treatments, clinical trials, virtual health such as telemedicine, etc., to facilitate the translation of knowledge generated from health and health services or infectious diseases studies into clinical practice and to inform health policy?
- For proposals addressing thematic priorities under Implementation Science (refer to Section 5b of the application), are the appropriate framework(s) / model(s) with the pre-set criteria proposed to evaluate/assess the barriers and facilitators of implementation outcomes clearly stated?
- For Seed Grant proposals (i.e. grant ceiling is HK$500,000), is the prospect that a successful outcome will enable scale-up to a larger project/trial and/or enhance the efficacy/effectiveness of existing practice clearly stated and feasible?

The proposed design and methodology for the study appear appropriate and well-justified. The study will use a multicenter, open-label, pilot randomized controlled trial to investigate the clinical utility of a ctDNA-guided approach for adjuvant chemotherapy in MIUC patients. The sample collection and processing methods are clearly described, including the use of STRECK tubes for blood collection and formalin fixation for tumor tissue. Whole exome sequencing will be used to analyze genetic profiling, including TMB, MSI, and HLA, and the sequencing will be performed by a reliable service provider. The sample size estimation appears to be realistic and justified. The proposed timeframe appears to be feasible, with patient recruitment planned over a 12-month period and follow-up scheduled for 24 months. The proposal acknowledges potential delays in recruitment due to the COVID-19 pandemic and provides contingency plans to address these challenges.

4. **Outcomes and Data Analysis** (i) Are the primary and secondary outcomes clearly defined? (ii) Have potential problems been anticipated and addressed? (iii) Is the statistical/analytical design appropriate and clearly explained?

the proposal provides clear definitions of primary and secondary outcomes, anticipates and addresses potential problems, and uses an appropriate statistical/analytical design.

5. **Research Capability** Comment on (i) the research team's expertise and track record (incl. principal investigator / project team members / collaborators) and (ii) the existing facilities of the Institution where the research will be conducted.

It is positive to see that the research team for the proposed study includes a range of professionals with diverse expertise, including urologists, oncologists, imageologists, epidemiologists, and research staff. The involvement of professionals from multiple disciplines can help to ensure that the study is designed and conducted in a comprehensive and rigorous manner, while also facilitating the translation of research findings into clinical practice.

6. **Budget** Is the request for research personnel, consumables, equipment and overall budget justified and reasonable? [For reference, 1 USD is equivalent to approximately 7.8 HKD]

the request appears to be justified and reasonable based on the information provided

7. **Ethical and Safety Considerations** Is the proposed research ethically sound? Outline any safety or ethical issues that from the proposed research and comment on whether these have been adequately addressed in the proposal. Has ethical approval been sought?

the proposal appears to be ethically sound, with appropriate consideration given to potential safety and ethical issues

8. **Overall Comments and Conclusion** It is always helpful for applicants to receive constructive feedback from reviewers. What are the specific strengths and weaknesses of this proposal? Please include a brief overall appraisal of the proposal focusing on any areas for improvement and the basis for your comments, e.g. awareness of other work in the field.

Strengths:

Overall, the proposal for a pilot randomized controlled trial investigating the clinical utility of a ctDNA-guided approach for adjuvant chemotherapy in patients with muscle-invasive urothelial carcinoma appears to be well-designed and justifiable. The proposal presents a clear rationale for the study, outlines appropriate methods and measures for evaluating the primary and secondary outcomes, and describes the use of appropriate statistical/analytical methods. The proposal also highlights the importance of this study for informing future clinical practice and research.

Strengths of the proposal include the use of whole exome sequencing and genetic profiling to assess MRD and predict patient outcomes, the involvement of a diverse and experienced research team, and the use of a randomized controlled trial design.

Weaknesses:

No

REFEREE'S ASSESSMENT FORM

Reference No.: 11221566

Project Title: Surveillance of the Genetic Signature in Circulating Tumor DNA for Guiding Adjuvant Chemotherapy in Urothelial Carcinoma: A Pilot Randomized Controlled Trial

PART A: REFEREE'S DETAILED REMARKS ON THE INDIVIDUAL SECTIONS OF THE GRANT APPLICATION

1. **Originality and Impact** What is the importance of the proposed research in terms of its originality and potential impact in the area under study? How will the research findings benefit patients and/or the healthcare system? Will the research findings improve patient care, population health, influence clinical practice and/or health services management, or inform health policy in Hong Kong and elsewhere? Have the potential facilitators and barriers to this impact being achieved been identified?

Overall, the proposed research project appears to be highly relevant and important in the field of urothelial cancer management. The study aims to assess the clinical efficacy of a ctDNA-guided approach for adjuvant chemotherapy in patients with urothelial cancer. While there is already some evidence to support the use of ctDNA-guided approaches in other cancer types, the proposed pilot RCT suggests that there is a gap in the literature when it comes to urothelial cancer.

2. **Research Questions, Aims and Hypotheses** How specific, clearly expressed and realistic are the research questions, aims and hypotheses?

The three aims are clearly expressed and focus on evaluating the clinical utility of ctDNA-guided approaches for adjuvant chemotherapy in urothelial cancer patients, assessing MRD surveillance through serial ctDNA testing, and providing preliminary data for a future phase III clinical trial. The two hypotheses presented are also clear and specific.

3. **Subjects and Study Methodology** (i) Is the proposed design and methodology appropriate for the study? (ii) Are sample sizes clear, justified, adequate and realistic? (iii) Are any preliminary data available? (iv) How feasible is the proposed timeframe? (v) Please also provide comments on the following (where applicable):

- For proposals submitted under Advanced Medical Research (refer to Area of Project on page 1 of application), is this a clinical study which applies advanced technologies including but not limited to biotechnology in medicine, use of drugs and treatments, clinical trials, virtual health such as telemedicine, etc., to facilitate the translation of knowledge generated from health and health services or infectious diseases studies into clinical practice and to inform health policy?
- For proposals addressing thematic priorities under Implementation Science (refer to Section 5b of the application), are the appropriate framework(s) / model(s) with the pre-set criteria proposed to evaluate/assess the barriers and facilitators of implementation outcomes clearly stated?
- For Seed Grant proposals (i.e. grant ceiling is HK$500,000), is the prospect that a successful outcome will enable scale-up to a larger project/trial and/or enhance the efficacy/effectiveness of existing practice clearly stated and feasible?

The proposed design and methodology for the study appear appropriate and well-justified. The study will use a multicenter, open-label, pilot randomized controlled trial to investigate the clinical utility of a ctDNA-guided approach for adjuvant chemotherapy in MIUC patients. The sample collection and processing methods are clearly described, including the use of STRECK tubes for blood collection and formalin fixation for tumor tissue. Whole exome sequencing will be used to analyze genetic profiling, including TMB, MSI, and HLA, and the sequencing will be performed by a reliable service provider. The sample size estimation appears to be realistic and justified. The proposed timeframe appears to be feasible, with patient recruitment planned over a 12-month period and follow-up scheduled for 24 months. The proposal acknowledges potential delays in recruitment due to the COVID-19 pandemic and provides contingency plans to address these challenges.

4. **Outcomes and Data Analysis** (i) Are the primary and secondary outcomes clearly defined? (ii) Have potential problems been anticipated and addressed? (iii) Is the statistical/analytical design appropriate and clearly explained?

The proposal provides clear definitions of primary and secondary outcomes, anticipates and addresses potential problems, and uses an appropriate statistical/analytical design.

5. **Research Capability** Comment on (i) the research team's expertise and track record (incl. principal investigator / project team members / collaborators) and (ii) the existing facilities of the Institution where the research will be conducted.

It is positive to see that the research team for the proposed study includes a range of professionals with diverse expertise, including urologists, oncologists, imageologists, epidemiologists, and research staff. The involvement of professionals from multiple disciplines can help to ensure that the study is designed and conducted in a comprehensive and rigorous manner, while also facilitating the translation of research findings into clinical practice.

6. Budget Is the request for research personnel, consumables, equipment and overall budget justified and reasonable? [For reference, 1 USD is equivalent to approximately 7.8 HKD]

The request appears to be justified and reasonable based on the information provided

7. Ethical and Safety Considerations Is the proposed research ethically sound? Outline any safety or ethical issues that from the proposed research and comment on whether these have been adequately addressed in the proposal. Has ethical approval been sought?

The proposal appears to be ethically sound, with appropriate consideration given to potential safety and ethical issues

8. Overall Comments and Conclusion It is always helpful for applicants to receive constructive feedback from reviewers. What are the specific strengths and weaknesses of this proposal? Please include a brief overall appraisal of the proposal focusing on any areas for improvement and the basis for your comments, e.g. awareness of other work in the field.

Strengths:

Overall, the proposal for a pilot randomized controlled trial investigating the clinical utility of a ctDNA-guided approach for adjuvant chemotherapy in patients with muscle-invasive urothelial carcinoma appears to be well-designed and justifiable. The proposal presents a clear rationale for the study, outlines appropriate methods and measures for evaluating the primary and secondary outcomes, and describes the use of appropriate statistical/analytical methods. The proposal also highlights the importance of this study for informing future clinical practice and research.

Strengths of the proposal include the use of whole exome sequencing and genetic profiling to assess MRD and predict patient outcomes, the involvement of a diverse and experienced research team, and the use of a randomized controlled trial design.

Weaknesses:

The preliminary data shown in Figure 3 contains only 3 patients. The ctDNA detection should be performed in more patients to inform the feasibility of this study.
